# Determining Phenological Patterns Associated with the Onset of Senescence in a Wheat MAGIC Mapping Population

**DOI:** 10.3389/fpls.2016.01540

**Published:** 2016-10-24

**Authors:** Anyela V. Camargo, Richard Mott, Keith A. Gardner, Ian J. Mackay, Fiona Corke, John H. Doonan, Jan T. Kim, Alison R. Bentley

**Affiliations:** ^1^National Plant Phenomics Centre, Institute of Biological Environmental and Rural Sciences, Aberystwyth UniversityAberystwyth, UK; ^2^UCL Genetics InstituteUniversity College London, UK; ^3^The John Bingham Laboratory, National Institute of Agricultural BotanyCambridge, UK; ^4^The Pirbright InstituteSurrey, UK

**Keywords:** wheat, senescence, data science, phenology, phenotyping, MAGIC

## Abstract

The appropriate timing of developmental transitions is critical for adapting many crops to their local climatic conditions. Therefore, understanding the genetic basis of different aspects of phenology could be useful in highlighting mechanisms underpinning adaptation, with implications in breeding for climate change. For bread wheat (*Triticum aestivum*), the transition from vegetative to reproductive growth, the start and rate of leaf senescence and the relative timing of different stages of flowering and grain filling all contribute to plant performance. In this study we screened under Smart house conditions a large, multi-founder “NIAB elite MAGIC” wheat population, to evaluate the genetic elements that influence the timing of developmental stages in European elite varieties. This panel of recombinant inbred lines was derived from eight parents that are or recently have been grown commercially in the UK and Northern Europe. We undertook a detailed temporal phenotypic analysis under Smart house conditions of the population and its parents, to try to identify known or novel Quantitative Trait Loci associated with variation in the timing of key phenological stages in senescence. This analysis resulted in the detection of QTL interactions with novel traits such the time between “half of ear emergence above flag leaf ligule” and the onset of senescence at the flag leaf as well as traits associated with plant morphology such as stem height. In addition, strong correlations between several traits and the onset of senescence of the flag leaf were identified. This work establishes the value of systematically phenotyping genetically unstructured populations to reveal the genetic architecture underlying morphological variation in commercial wheat.

## Introduction

Wheat is a pillar of global food security, providing 20% of protein and calories consumed worldwide and up to 50% in developing countries. It is the main food staple in Central Asia, West Asia and North Africa, which have the world's highest per capita wheat consumption (Valluru et al., 2015). Global wheat production is at risk due to climate change, population growth, changing food preferences and the plant health challenges associated with its widespread cultivation. In order to maintain optimal production and profitability, wheat producers and processors must prepare for and adapt to these challenges. The current emphasis on food security has focused research attention on two avenues to improve wheat yield (Valluru et al., 2015): (1) increasing photosynthetic capacity and efficiency (Reynolds et al., 2009); and (2) increasing partitioning of assimilates to the developing spike and grain.

The timing of key developmental transitions is critical for many crops, but is particularly important in the temperate small grain cereals. For example, the transition from vegetative to reproductive growth can have major effects on biomass accumulation and harvest index that profoundly affect either the locales in which a variety can be profitably grown, or its ultimate use. Thus, crops destined for grain production should transition early, relative to the length of the growing season, to allow ripening, avoid stress, and achieve a high harvest index of grain to total biomass. Forage, biofuel or dual purpose crops could usefully transition later to allow greater total biomass accumulation, but this has to be tempered with the likelihood of deleterious stress/weather events. Flowering time, therefore, has been a key selection target since the beginning of domestication (Izawa, 2007), initially inadvertently but since modern breeding began, very directly.

Understanding the extent and basis of other aspects of phenological variation may be useful in breeding for yield potential and stress adaptation. In wheat, leaves can contribute up to 40% of the nitrogen incorporated by the grains on the fifteenth day after anthesis (Simpson et al., 1983). Therefore, lifespan of the leaves (hence yield) is a trade-off with N remobilization. Delayed leaf senescence (as in the stay-green effect), which maintains active photosynthesis for a longer period, can increase grain yields under certain circumstances (Gregersen et al., 2008). Conversely, accelerated senescence leads to low carbon (C) but high N remobilization, indicating plasticity of C and N remobilization during development, perhaps correlated with senescence. A better understanding the genetic and environmental factors affecting these processes would help optimize C and N remobilization to the actively developing grains under different growth/stress conditions.

**Figure 1:**
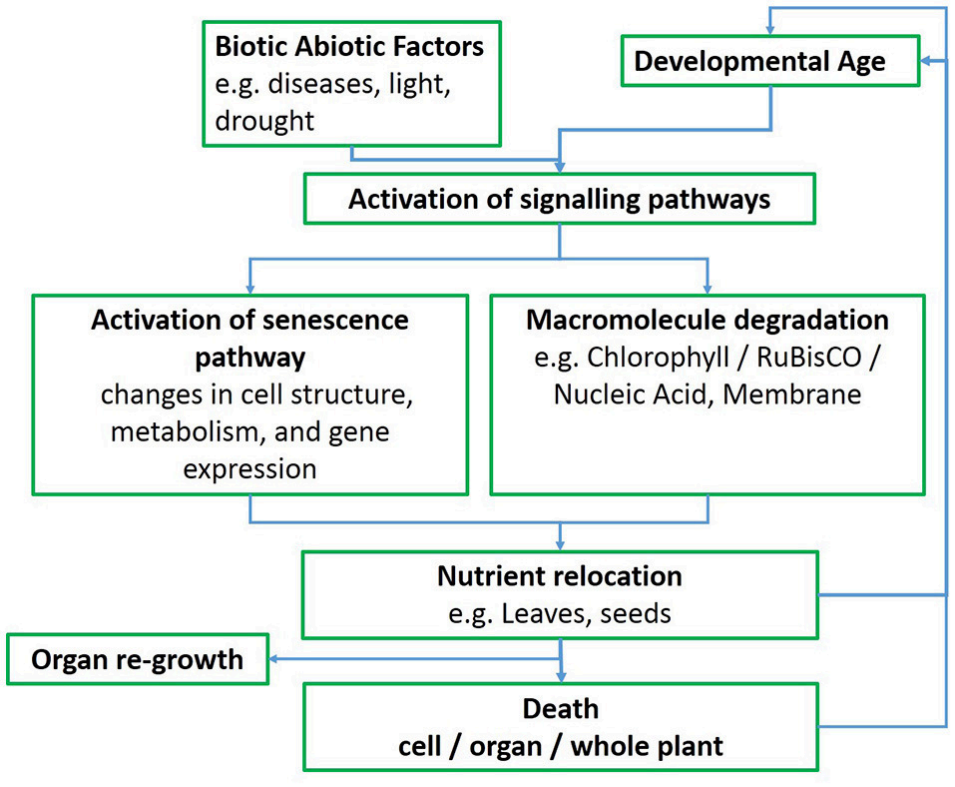
**Whole leaf senescence flowchart**.

While several patterns of senescence have been proposed (Thomas and Howarth, 2000), an ideal senescence phenotype in wheat, and in cereals in general, still needs to be identified (Gregersen et al., 2008), perhaps because of strong and variable environmental effects. In monocarpic crops such as wheat the initiation of senescence typically leads to a massive remobilization of phloem-mobile nutrients from the senescing plant parts to developing sinks, such as seeds or grains (Figure 1; Gregersen et al., 2008; Distelfeld et al., 2014). Pathogen infection also interacts with developmental processes in a complex way and symptoms of senescence often accompany the progression of disease, although senescence can also be delayed in response to pathogen infection (Häffner et al., 2015).

A major constraint to progress in breeding for high yield varieties is the access to appropriate and consistent selection environments. The selection environment plays a key role in the efficiency of the selection process. Since environmental variables are almost impossible to control under field conditions, the identification of specific genetic factors associated to crop yield becomes more challenging (Bentley et al., 2013).

Modern controlled environment (CE) growth control and/or recording of variable environmental parameters such as temperature and watering allow for elimination or reduction of uncontrollable influences. Having control and access to an experiment's environmental parameters allows for reproducibility, and decreases the levels of uncertainty as it is easier to reverse-engineer an experiment in order to identify—or at least justify—the causes of a given phenotype, and minimizes the amount of replication per subject due to the low variability of the environment. Therefore, CE phenotyping offers closely defined conditions compared to the relatively homogeneous but less controllable growing conditions in a field plot.

To understand the genetic control of phenology and the onset of senescence in wheat, we screened a core set of the NIAB elite MAGIC wheat population (Mackay et al., 2014) across time. The eight founders of this MAGIC population were selected in partnership with UK wheat breeders to sample trait variation and germplasm important to current UK breeding programmes (Bentley et al., 2013). MAGIC populations combine high levels of genetic diversity, recombination and homozygosity (Mackay et al., 2014) to create a panel of recombinant inbred lines (RILs). A well-designed MAGIC population captures and immortalizes the variation released by intercrossing, thereby providing a stable well-defined population to be shared across sites and used across years.

In this study, plants from the elite MAGIC core set and its parents were scored throughout their life cycle to capture traits, including decimal growth stages, biomass and plant height. We discuss the use of a subset of MAGIC lines in the Smarthouse as a proof of concept that the combination of MAGIC + Smarthouse phenotyping should be repeated on a grander scale.

## Materials and methods

### Plant material

A subset of the NIAB Elite eight-founder MAGIC population described in Mackay et al. (2014) was used for all phenotypic screening. The complete population consists of approximately 1000 recombinant inbred lines (RILs) generated from three cycles of recombination between eight elite United Kingdom wheat varieties (Alchemy, Brompton, Claire, Hereward, Rialto, Robigus, Soissons, Xi-19) followed by five rounds of selfing to derive RILs. Further information about the population, including pedigree, genotype, and existing phenotype data can be found at www.niab.com/MAGIC.

The core set used in this study was selected to represent all funnels of the 210 8-way crosses within the population. Two funnels were not represented due to limited seed availability making a total of 208 RILs in the core set.

### Glasshouse cultivation

Plants were grown between mid-January 2015 and mid April 2015 in The National Plant Phenomics Centre facilities in Aberystwyth, UK. The eight parents of the MAGIC population and four additional elite varieties (Avalon, Santiago, Cadenza, and Zircon) were grown with the 208 RILs (see Table S1) under well watered conditions, with two replicates per genotype. Two seeds were sown in 8 × 8 cm pots of Levington F2 compost. After germination (approximately on the 30/10/2014) the seedlings were thinned to one per pot and transferred to a controlled environment room for vernalization (5°C, 16 h daylength) for 9 weeks. Following vernalization plants were transferred to 15 × 15 × 20 cm pots of M2 compost. Field capacity and dry matter content of the compost was determined. Plants were transferred to the growth chamber where each pot was placed into a cart on a conveyor system. Pots were weighed and watered automatically to 75% gravimetric water content daily. Growth conditions were 14 h daylength using 600W sodium lamps to supplement (350 μM/m^2^/sec) natural lighting, with the temperature settings of 18°C (day) and 15°C (night). Plant hygiene was monitored by visual inspection throughout the experiment, with an appropriate prophylactic and responsive spraying regime. Once the ears started to ripen, plants were removed from the system and allowed to finish ripening naturally with reduced watering. Reduced watering only occurred after all plants had passed Flag leaf senescence.

### Phenotyping

Plants were manually scored for developmental stages according to the Zadoks scale (Zadoks et al., 1974) three times per week, and scored as days after sowing (DAS) when plants reached growth stage 39 (GS39; flag leaf fully emerged), GS55 (ear 50% emerged), GS65 (50% anthesis), and the onset of flag leaf senescence (FLS). At the end of the experiment, plants were harvested and above ground biomass (PW), tiller number (TN), plant height (PH), stem height (SH), top internode length (TIL), first/second/third ear length (FEL, SEL, TEL), first/other ear weight (FEW/OEW), and first flag leaf length (FFLL) were scored. The number of days between GS39 and GS55 (d1), GS55 and GS65 (d2) and GS55 and FLS (d3) were also determined. A multiple linear regression model (MLRM) was fitted to identify predictors of FLS among all the traits used in the analysis. A list of traits, abbreviations and Crop ontology terms (Shrestha et al., 2012) are provided in Table 1.

**Table 1:** **Plant trait descriptions**.

**No**.	**Abbreviation in paper**	**Trait description (wheat ontology)**	**Unit**	**Wheat ontological abbreviation**	**Trait ID (TO or CO)**
1	GS39	Growth/developmental stages based on zadoks decimal code: Flag leaf fully emerged	DAS^*^	GrwStg Zadok 39	CO_321:0000016
2	GS55	Growth/developmental stages based on zadoks decimal code: Ear 50% emerged	DAS	GrwStg Zadok 55	CO_321:0000016
3	GS65	Growth/developmental stages based on zadoks decimal code: Anthesis time	DAS	GrwStg Zadok 65	CO_321:0000016
4	FLS	Onset of flag leaf senescence. FLS is scored on the day after sowing when greater than 2.5 cm at the tip the primary flag leaf was senescent (color changed from green to yellow/brown)	DAS	tFleafSen	TO:0000249
5	PW	All above-ground biomass at maturity	g/plant		CO_321:0001431
6	TC	Tiller number	Tiller/plant		CO_321:0000190
7	SH	Stem height (it is stem height to the base of the ear for primary tiller)	cm		CO_321:0000060
8	TIL	Top internode length	cm		TO:0000145
9	FEL	First (primary) ear length	cm	SpkLng	CO_321:0000056 TO:0000431
10	SEL	Second ear length	cm	SpkLng	CO_321:0000056 TO:0000431
11	TEL	Third ear length	cm	SpkLng	CO_321:0000056 TO:0000431
12	FEW	First (primary) ear weight	g		n/a
13	OEW	Other ear weight	g		n/a
14	TEW	Total ear weight (FEW + OEW)	g		n/a
15	FFLL	First (primary) flag leaf length	cm	FLFLG	TO:0002757
16	d1	Number of days between GS39 and GS55	Days		n/a
17	d2	Number of days between GS55 and GS65	Days		n/a
18	d3	Number of days between GS55 and FLS	Days		n/a
19	SM	Plant stress score mean	Observational score		n/a
20	HI	Harvest Index	Ratio	HI	TO:0000128

*TO, Trait Ontology (Liang et al., 2008)*.

*CO, Crop Ontology (Shrestha et al., 2012)*.

* *DAS, Days after sowing*.

*Wheat ontology (Leo Valette, Bioversity, France, Personal Communication)*.

*Unit is the metric of thetrait. Trait id is the TO or CO reference id*.

### Genotyping

The lines were genotyped using the Illumina Infinium iSelect 80,000 SNP wheat array (“80K array,” http://www.illumina.com/), described in Wang et al. (2014). 20,639 SNP markers were scorable and polymorphic, of which 18,601 were successfully mapped in the MAGIC population (Mackay et al., 2014; Gardner et al., 2016). Linkage map generated by mpMap is reported in Gardner et al. (2016).

### Plant stress

The young plants showed symptoms that included chlorosis and necrosis (Figure S1). The chlorotic symptoms consisted of yellow areas surrounding lesions on the leaf blades. The necrotic symptoms comprised brown spots, lens-shaped lesions, surrounded by yellow borders. Although symptoms were controlled by routine spraying (Priori Xtra, Syngenta), we speculated that it constituted an undiagnosed disease (possibly Septoria) and the degree of infection was scored manually using the seedling infection type (IT) score shown in Table S1. Plant visual stress symptoms were scored first at GS31–39 and for the second time around GS70–GS80, and the average was calculated (SM). These qualitative IT scores were converted to a numerical scale for statistical analysis.

### Statistical and quantitative trait locus analysis

Statistical analyses were performed in the R environment using Core Team (2013). Quantitative Trait Locus (QTL) analysis was performed using the R package HAPPY for multi-parental populations analyses (Mott et al., 2000). The genetic analysis of multi-parental populations requires a haplotype-based approach because single marker association or interval mapping can fail to detect a QTL if the causative alleles are not dispersed among the founders with the same strain distribution pattern as the linked markers (Mott et al., 2000).

### QTL mapping

HAPPY's analysis is essentially two stage; ancestral haplotype reconstruction using dynamic programming, followed by QTL testing by linear regression:

- Assume that at a QTL, a pair of chromosomes originating from the progenitor strains, labeled *s, t* contribute an unknown amount *T*_*st*_ to the phenotype. In the special case where the contribution from each chromosome is additive at the locus then *T*_*st*_ = *T*_*s*_ + *T*_*t*_, say
- A test for a QTL is equivalent to testing for differences between the *T*'s.
- A dynamic-programming algorithm is used to compute the probability *F*_*iLst*_ that a given individual *i* has the ancestral alleles *s, t* at locus labeled *L*, conditional upon all the genotype data for the individual. Then the expected phenotype is
  $$\begin{array}{l} y=\underset{st}{\sum }T_{st}F_{iLst}, \end{array}$$
  and the *T*'s are estimated by a linear regression of the observed phenotypes on these expected values across all individuals, followed by an analysis of variance to test whether the progenitor estimates differ significantly.
- The method's power depends on the ability to distinguish ancestral haplotypes across the interval.
- All inference is based on regression of the phenotypes on the probabilities of descent from the founder loci, *F*_*nst*_.

The models are presented here in the linear model framework (i.e., least-squares estimation, with ANOVA *F*-tests).

For an additive QTL, the parameters are the strain effect sizes; for a full interaction model there is a parameter for every possible strain combination. Then the one-QTL model is *E*(*y*) = *X*_*L*_*t*_*L*_.

There are *S*(*S* − 1)/2 + *S* parameters (where *S* is the number of strains) to be estimated in a full model allowing for interactions between the alleles within the locus, and *S* − 1 parameters in an additive model. For the full model, the *i, j*'th element of the design matrix *X* is related to the strain probabilities thus:

$$\begin{array}{l} X_{Lij}=F_{iLst}, \end{array}$$

where

$$\begin{array}{l} \left(s,t\right)=in\left(s+S\left(t-1\right)\right),t+S\left(s-1\right) \end{array}$$

and for the additive model

$$\begin{array}{l} X_{Lij}=\underset{s}{\sum }F_{iLsj} \end{array}$$

We used an additive model, where the contribution of each allele at the locus are assumed to act additively.

Furthermore, when mapping QTLs in structured populations the evidence for the existence of a QTL has to be considered in the context of other QTLs, which might explain some of the same component of variation. Population structure can produce long-range correlations between genotypes and hence ghost QTL, although the LD analysis suggests that the MAGIC population is relatively immune to this phenomenon. Although the MAGIC population is relatively unstructured, and therefore can be analyzed one locus at a time, in order to ensure the evidence for a given QTL was not confounded with that for others, statistical significance was assessed based on permuting the phenotypes (1000 times) between individuals, repeating the model fit, and finding the top-scoring marker interval. The empirical distribution of the max −logP values was then used to assess statistical significance. This technique is useful for non-normally distributed phenotypes and for estimating region-wide significance levels. We used −logP = 4 as a threshold in the multiple QTL modeling to test for association and FDR = 0.05 to identify significantly differential markers. The dashed line in QTL plots corresponds to an FDR rate of 0.05 and is calculated using the qvalue package (Storey and Tibshirani, 2003). The *p*-value corresponding to a *q*-value of 0.05 is determined by interpolation. When there are no *q*-values less than 0.05, the dashed line is omitted.

In addition, a multiple linear regression model (MLRM) was fitted to identify predictors of FLS among all the traits used in the analysis. We selected FLS because the trait is used as indicator of crop yield and biomass accumulation (Gan, 2014).

Principal Component Analyses (PCA) over normalized trait data was carried out to identify patterns between traits and genotypes. Biplots were used to show information on samples in a graphical manner (Kempton, 1984). PCA of marker data was carried out separately to test for population structure.

### QTL validation

We used the R package mpMap to confirm QTL mapping results (Huang and George, 2011) and to analyse the effect of including marker covariates. QTL analysis is performed using interval mapping, then selected marker covariates are included in the linear model in a forward selection process.

### Data processing

Data were pre-processed using standard methods. Data corresponding to one replicate of the MAGIC line MEL 086-1 (note, all MAGIC lines are named with prefix “MEL”) and another from one replicate of the elite line Avalon were removed due to seed infection. A small number of outliers (data points with suspicious values) were checked and, where possible, corrected. Missing values (2.19%) were imputed using multivariate imputation by chained equation (MICE) (van Buuren and Groothuis-Oudshoorn, 2011). Briefly, MICE operates under the assumption that given the variables used in the imputation procedure, the missing data are Missing At Random (MAR), which means that the probability that a value is missing depends only on observed values and not on unobserved values (Schafer and Graham, 2002). MICE creates a number of datasets by imputing missing values. That is, one missing value in original dataset is replaced by m plausible imputed values. We set *m* = 5 as the number of imputations. These values take imputation uncertainty into consideration. Statistics of interest are estimated from each dataset and then combined into a final one and replicates were averaged (Zhang, 2016).

## Results

**Figure 2:**
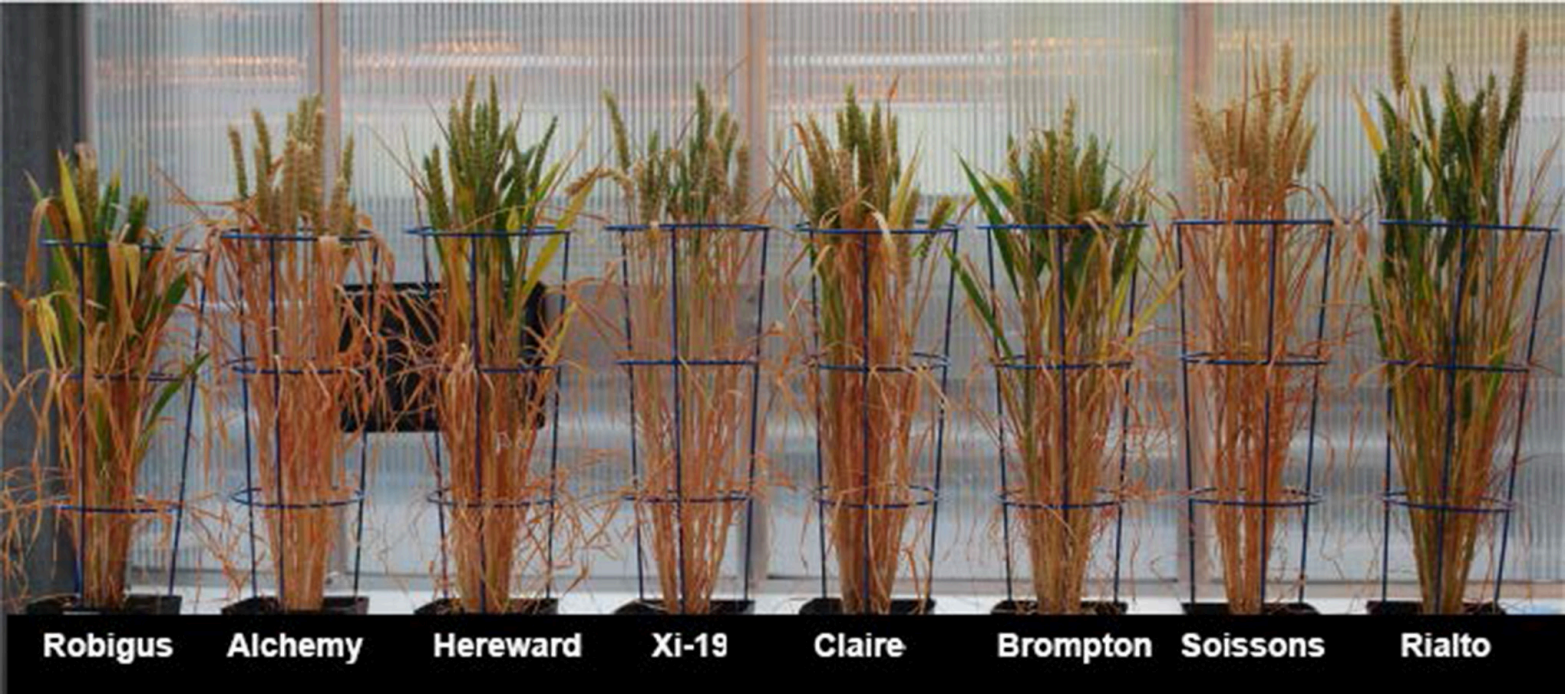
**MAGIC parents at senescence**.

In this experiment, RILs, MAGIC parents (illustrated in Figure 2) and 4 other elite genotypes were grown to maturity over a single time span and within a single glasshouse chamber with controlled watering and supplementary lighting and heating. Phenotype data were curated, and missing values (accounting for 2.19% of data points) imputed. Figure S2 shows a comparison between original data (red dots) and imputed data (blue dots), which suggested a high similarity between the two distributions as indicated by the overlapping dots.

### Analysis of traits

**Figure 3:**
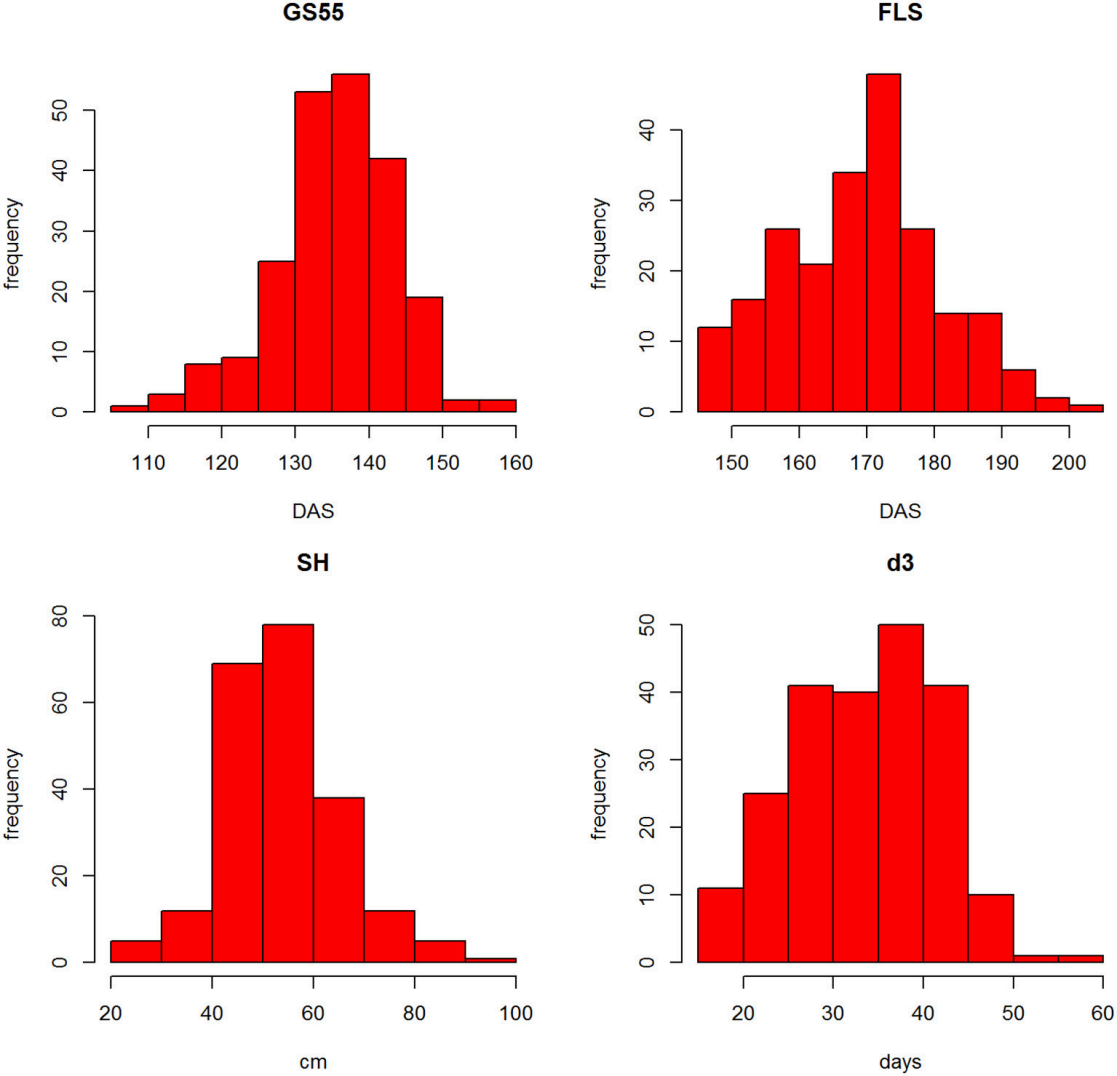
**Frequency distributions of GS55, FLS, SH, and d3 from all RILs**.

The distributions of the traits are shown in Figure S3. Most traits showed similar distributions with the exception of the discrete trait SM, which was skewed (see Table 1 for trait description). SM is a discrete trait and the skewedness of the plot reflects that most RILs' scores were in the 0–2 range. Figure 3 shows the frequency distributions for GS55, FLS, SH and d3. Pair-wise correlation analysis between all traits (Figure S3) identified strong correlations between FLS and GS39 (0.79), GS55 (0.73) and GS65 (0.69) and d3 (0.70); between FEL, SEL, and TEL (>0.86) between TIL and SH (0.76), PW and OEW (0.87), and between TEW and PW (0.87).

**Figure 4:**
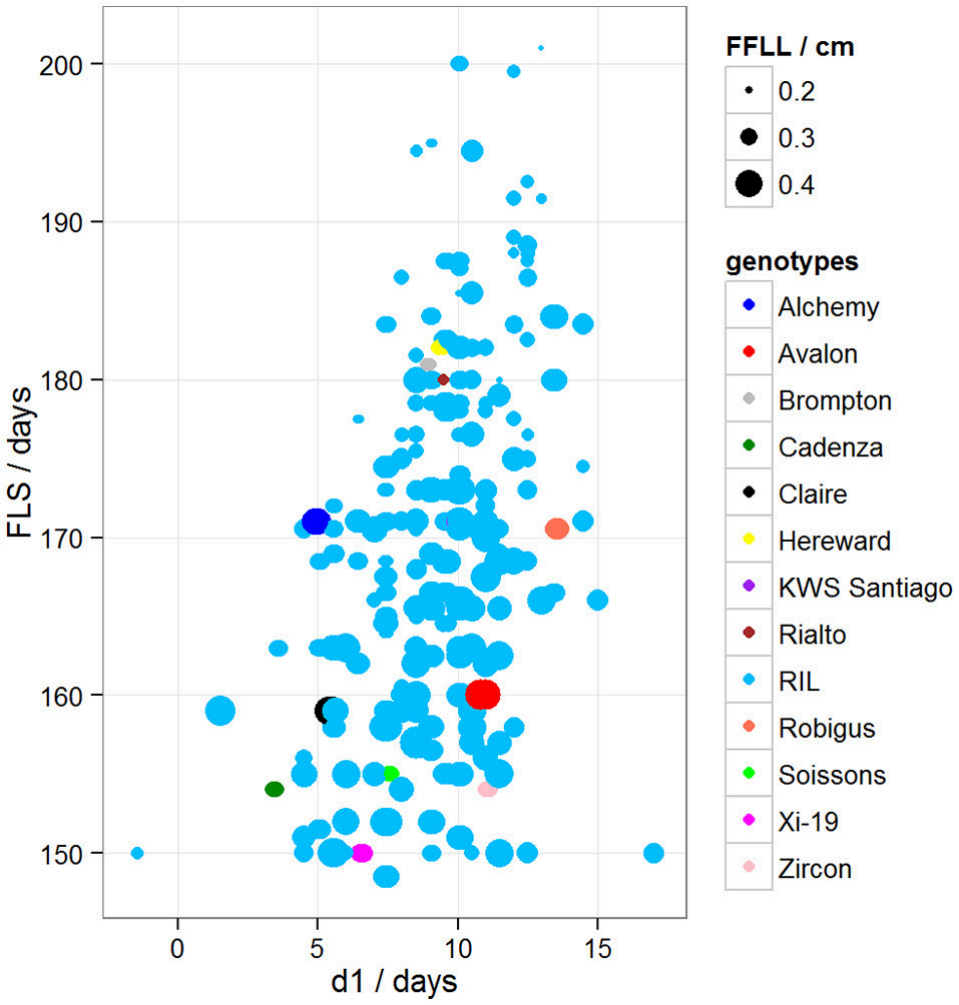
**Variation between d1 and FLS**. Dot size represents FFLL/100 (divided by 100 to be able to show the dots in the plot). Blue dots correspond to RILs. Other colored dots correspond to MAGIC parents.

**Figure 5:**
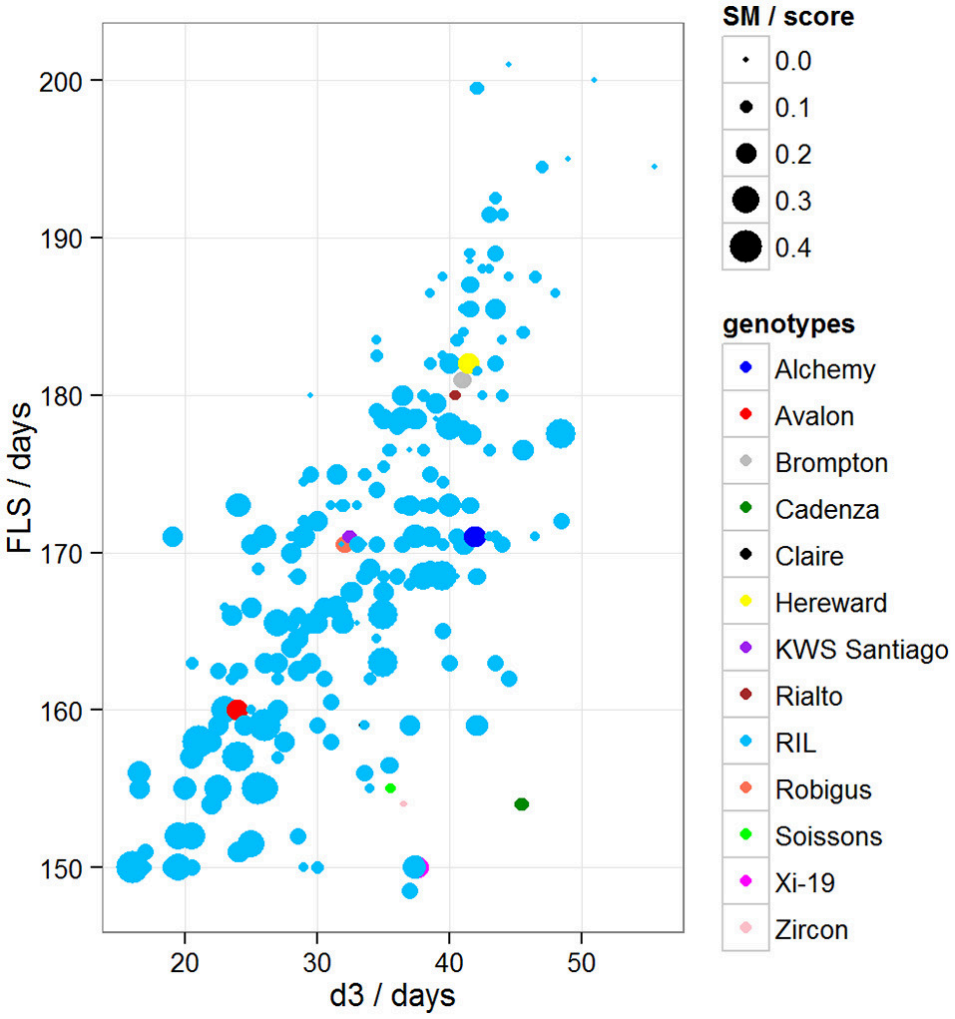
**Variation between d3 and FLS, where d3 = days between GS55 and FLS**. Dot size represents disease score/10. Blues dots correspond to RILs and other colored dots to MAGIC parents.

To determine if there was variation in duration between key developmental stages that was not simply a result of variation in overall developmental progression, we examined the time in days taken to progress from GS39 to GS55 (d1), from GS55 to GS65 (d2) and from GS55 to FLS (d3). Our MLRM identified d3 as a strong predictor (*P* < 0.05) of FLS, indicating that the time lapse between GS55 and FLS is a good candidate to predict FLS (Figure 4). In addition to d3, our MLRM identified other important predictors of FLS (*P* < 0.05). For example, the size of the flag leaf on the primary shoot, FFLL, was significantly (*P* < 0.05) associated with the timing of senescence (FLS). To demonstrate this result, dot size in Figure 4 was used to represent an additional feature in the plots. In the case of Figure 4, FFLL (represented by dot size) was longer in RILs that senesced earlier. Among the MAGIC founders, Brompton, Hereward and Rialto senesced after Xi-19 and the two elite controls Zircon and Cadenza. The latter also had the shortest duration between GS39 and GS55. Previously, Mackay et al. (2011) reported Cadenza as the most environmentally sensitive variety detected in 8 years in Recommended List trials showing a linear increase in yield with increasing summer rainfall. This supports the observations from the trait analysis that progression through different developmental processes e.g., flowering vs. senescence, is controlled independently. Figure 5 shows d3 in relation to FLS (the contract of d2 to FLS is shown in Figure S5). Plants which senesced earlier took less time between GS55 and GS65 (d2) and between GS55 and FLS (d3).

**Figure 6:**
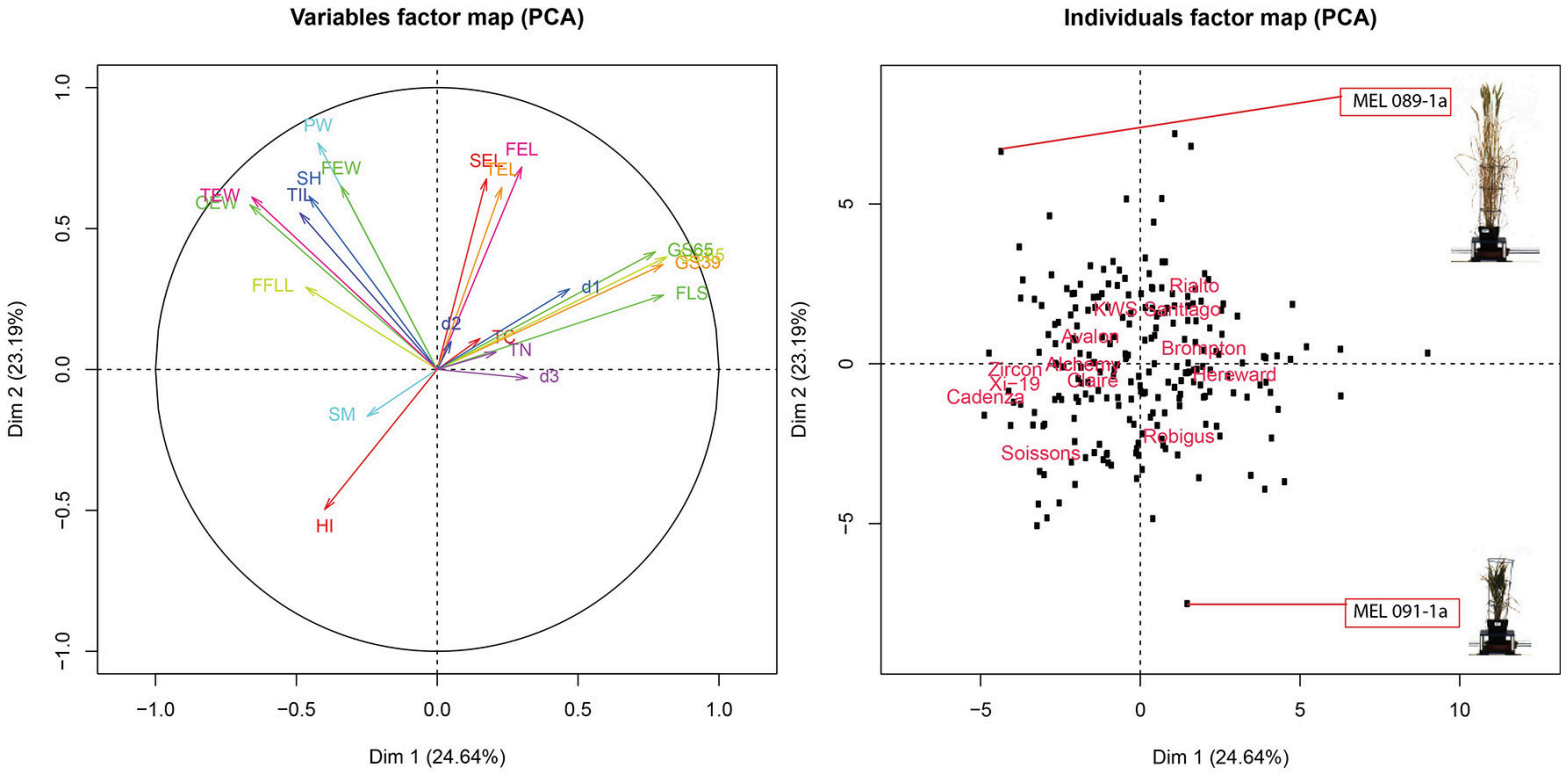
**(Left panel)** PCA score biplot for 18 different traits. **(Right panel)** Biplot corresponding to 8 MAGIC parent, 4 elite lines and 208 RILs. MAGIC parents are labeled only to facilitate interpretation.

To further evaluate the relationship between these traits, PCA was conducted over trait data scaled to have unit variance. Results of the analysis are shown on the biplot in Figure 6. The plot shows that PC1 and PC2 account for 48% of the total variance of the traits. Also, four clearly defined trait groups (anti clockwise) can be seen in the plot, the first one containing FLS, GS39, GS55 and GS65, d1, d3, and TN, the second group contained SEL, FEL and TEL, the third group contained OEW, PW, TIL, SH, FEW, and FFL and the fourth group contained HI and SM. Since (1) the smaller the angle between the trait vectors, the higher the correlation (2) trait values are smaller toward the middle of the plot and higher toward the edge, we can deduce that SM is negatively correlated to group one which is confirmed by the results from the correlation shown in Figure S4. The same argument could also be used between group one and group three, which is also confirmed by Figure S4.

Looking at the relationship between traits and MAGIC parents, Brompton and Hereward are positively correlated, tending to be slow to FLS (late to senesce) as indicated by their proximity to the FLS vector. In contrast, Soissons and Xi-19 show an opposite effect, rapidly reaching FLS as indicated by their location at the opposite side of FLS. Through this analysis we can also see that, in general, most RILs and parents have a similar overall phenome, as represented by their location close to the center of the plot. We can also see there are a number of divergent phenotypes, such as the one at the bottom of the plot (Figure 6, right panel) which corresponds to MAGIC line MEL 091-1a or the one at the top which correspond to MEL 089-1a. Looking closer, MEL 089-1a is proximal to PW and SH while MEL 091-1a is further away, which indicates these two lines contrast strongly for these particular traits. A picture of both lines taken on 27/04/2015 was added to the plot to facilitate interpretation. The plants show clearly contrasting differences in height and biomass.

### Plant stress analysis

A low level of chlorotic and necrotic lesions was observed on the leaves early during the growth period. Symptoms (SM) were scored independently by two people at two time points and scores were averaged. When comparing symptom scores against the onset of FLS, we found that the more severely affected plants started senescence earlier than those plants that were mildly affected (Figure 5). Our MLRM also identified SM as a predictor of FLS (*P* < 0.05).

Figure 6 confirms the negative correlation between FLS and SM as indicated by its opposite location from FLS. This correlation is consistent with that of *Mycosphaerella graminocola*, where infection induces senescence by manipulating signaling pathways in plants (Mengiste, 2012). However, it should be noted that the precise identity of the putative pathogen could not be confirmed.

### Quantitative trait loci

We evaluated whether trait variation could be ascribed to underlying genetic variation. The lines were genotyped using the Illumina Infinium iSelect 80,000 SNP wheat array (“80K array,” http://www.illumina.com/), described in Wang et al. (2014). 20,639 SNP markers were scorable and polymorphic, of which 18,601 were successfully mapped in the MAGIC population (Mackay et al., 2014; Gardner et al., 2016); linkage map for this population was produced using mpMap and reported in Gardner et al. (2016). First, we checked for signs of population structure. To do this, the marker based relationship matrix (A) was calculated using the R package rrBLUp (Endelman, 2011), then a PCA analysis by eigenvalue decomposition of A was calculated. Results are shown in Figure S7. This shows that the first PC accounts for less than 4% of the total spectrum. This confirms the expected absence of population structure. Genome mosaics corresponding to MEL 15-2, MEL 091-1a, and MEL 209-1 are shown in Figure S8. These decompose the lines' genomes into mosaics of founder haplotypes. The lines appear to be a random mix of the founders, which indicates an absence of gross population structure.

**Figure 7:**
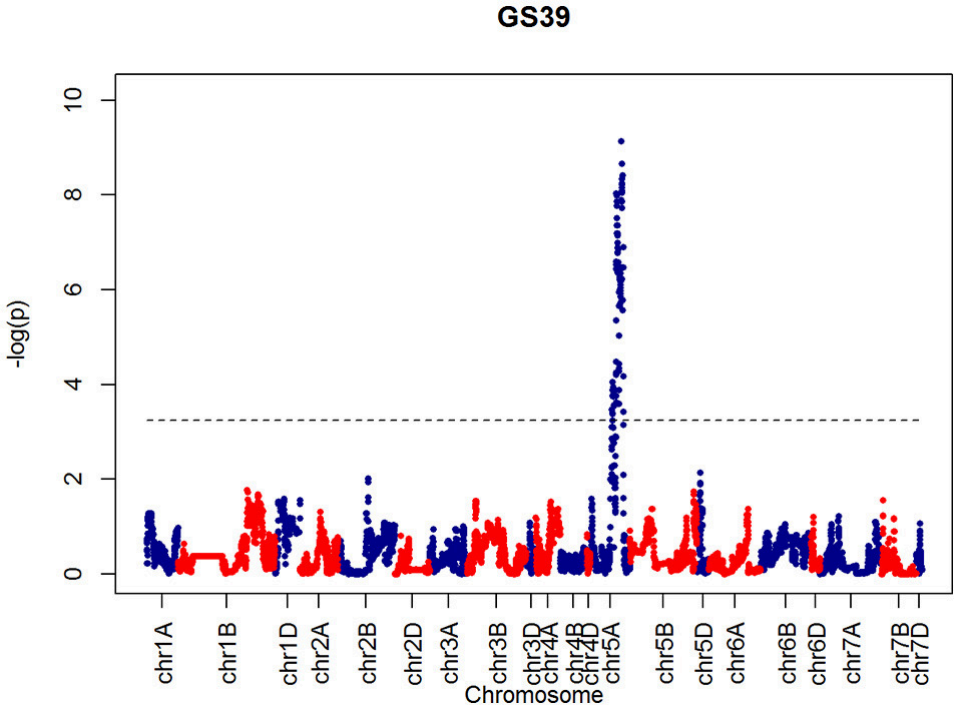
**QTLs markers for the trait GS39**. Dashed lines indicate threshold (FDR < 0.05, logP4).

**Figure 8:**
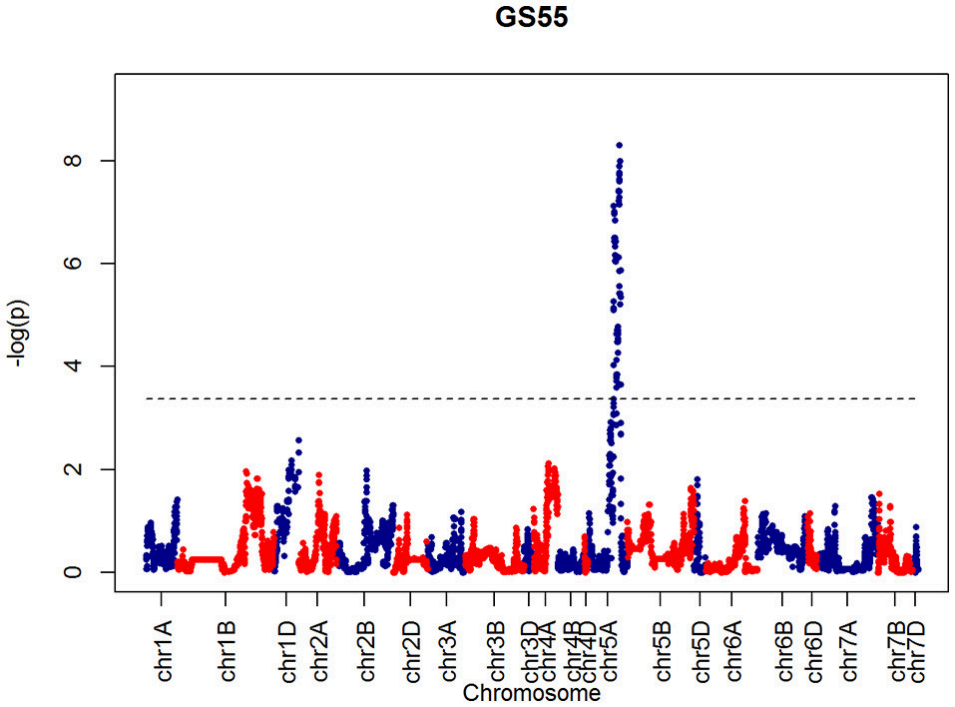
**QTLs markers for the trait GS55**. Dashed lines indicate threshold (FDR < 0.05, logP4).

After confirming the absence of population structure, we used HAPPY (Mott et al., 2000) to test for association between each phenotype and the predicted founder haplotypes at each locus in the genome. We used the *P*-value threshold < 10^−4^ to call QTLs [−logP = 4, corresponding to a false discovery rate (FDR) = 0.05]. This analysis identified loci associated with two phenological traits, GS39 and GS55, and a number of traits such as SEL, SH, TIL, TEW, and HI. For GS39, three significant QTLs were found on chromosome 5A, at 201.36, 212.52, and 224.64 cM, corresponding to the markers BS00009369_51, BS00021942_51 and wsnp_Ex_c5978_10478584, respectively (Table 2, Figure 7). For GS55, three QTLs were found on chromosome 5A, at 201.36, 216.05 and 227.66 cM, which correspond to BS00009369_51, wsnp_Ex_rep_c66689_65011117, and Excalibur_c7729_144, respectively (Figure 8). In both cases, these three close peaks are likely to represent a single QTL. To confirm this hypothesis, we performed composite interval analysis using 5A as covariate and identified a single clear and strong marker on 5A (Figures S11A–E).

**Table 2:** **QTLs mapped for different traits**.

**Phenotype**	**Markers**	**Chr**	**cM**	**−logP**	**h^2^**	**GW *P*-value**	**Alchemy**	**Brompton**	**Claire**	**Hereward**	**Rialto**	**Robigus**	**Soissons**	**Xi-19**
GS39	BS00009369_51	5A	201.36	8.04	0.35	0	127.34	127.15	125.21	128.42	128.01	125.13	127.07	111.46
GS39	BS00021942_51	5A	212.52	4.44	0.24	0.06	126.75	127.32	125.39	127.76	129.01	125.5	126.79	116.98
GS39	wsnp_Ex_c5978_10478584	5A	224.64	9.14	0.38	0	126.54	127.01	126.27	127.48	128.24	125.8	126.92	112.83
GS55	BS00009369_51	5A	201.36	7.13	0.33	0	136.79	135.89	134.09	138.57	136.96	134.49	136.99	119.18
GS55	wsnp_Ex_c37943_45584325	5A	216.05	4.64	0.25	0.03	135.3	135.57	134.37	138.38	137.99	134.57	136.39	127.6
GS55	Excalibur_c7729_144	5A	227.66	8.32	0.36	0	135.6	135.43	135.9	137.54	137.82	135.01	136.59	120.31
HI	Kukri_c27309_590	2D	55.4	4.97	0.01	0.01	0.55	0.55	0.55	0.55	0.55	0.55	0.6	0.55
OEW	RAC875_c6922_291	4D	26.97	4.09	0.21	0.06	37.4	37.14	37.17	36.3	36.81	42.6	39.2	35.57
PW	RAC875_c1673_193	4D	32.24	4.79	0.24	0.02	70.96	71.2	71.48	71.33	68.74	88.5	82.52	71.26
SEL	wsnp_Ex_c6548_11355524	5B	60.65	3.99	0.22	0.09	11.2	11.18	11.21	11.18	11.16	11.18	10.45	10.02
SEL	BS00001101_51	5B	66.38	4.01	0.22	0.09	11.12	11.19	11.19	11.13	11.17	11.33	10.36	10
SEL	wsnp_Ku_c2185_4218722	5B	90.8	4.15	0.23	0.07	11.08	11.16	11.1	11.08	11.14	11.48	10.36	10.2
SEL	RAC875_c19099_434	5B	92.31	3.97	0.22	0.09	11.05	11.12	11.05	11.1	11.02	11.27	10.49	10.3
SH	RAC875_c1673_193	4D	32.24	9.52	0.38	0	49.88	51.63	49.92	50.13	49.98	63.89	60.82	47.34
TEW	RAC875_c6922_291	4D	26.97	4.02	0.085	0.08	41.14	40.41	40.93	40.15	40.66	46.71	43.58	39.47
TIL	RAC875_c1673_193	4D	32.24	7.09	0.31	0	27.78	28.2	27.8	27.59	27.64	32.11	32.4	26.25

*cM is the marker position. −logP is −log10 at the QTL peak; h^2^ is the fraction of variance accounted for by QTL, after removing covariates Chr is the chromosome. P-value is the genome wise P-value for the QTL based on permutations. Alchemy, Brompton, Claire, Hereward, Rialto, Robigus, Soissons*.

**Figure 9:**
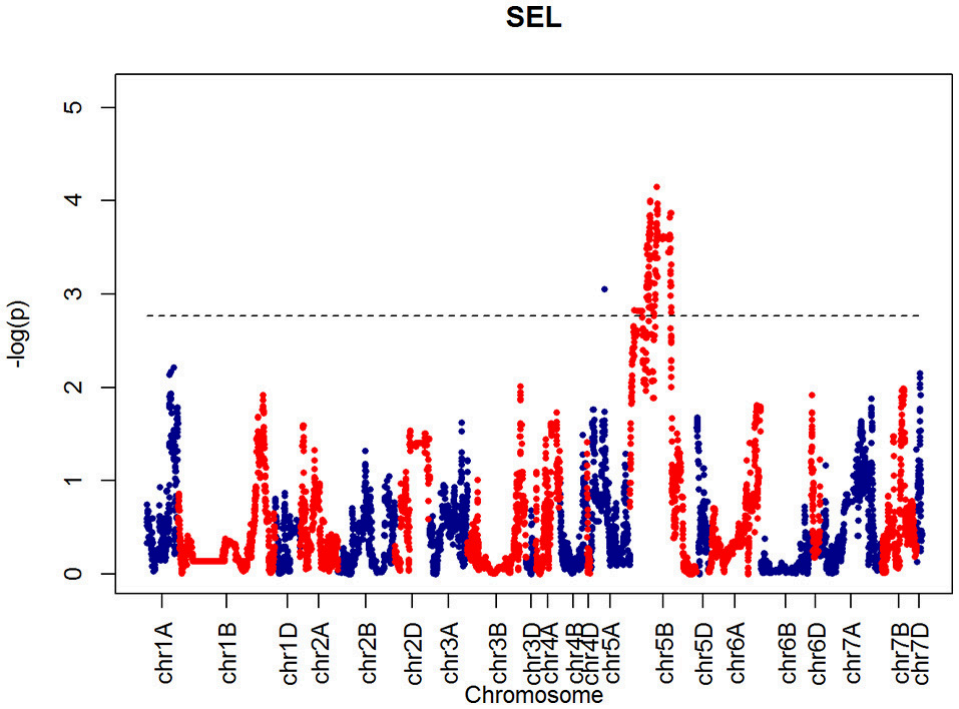
**QTLs markers for the trait SEL**. Dashed lines indicate threshold (FDR < 0.05, logP4).

For SEL, four QTLs were identified on chromosome 5B at 60.65, 66.38, 90.8, 92.31 cM, corresponding to wsnp_Ex_c6548_11355524, BS00001101_51, wsnp_Ku_c2185_4218722, and RAC875_c19099_434, respectively (Figure 9).

**Figure 10:**
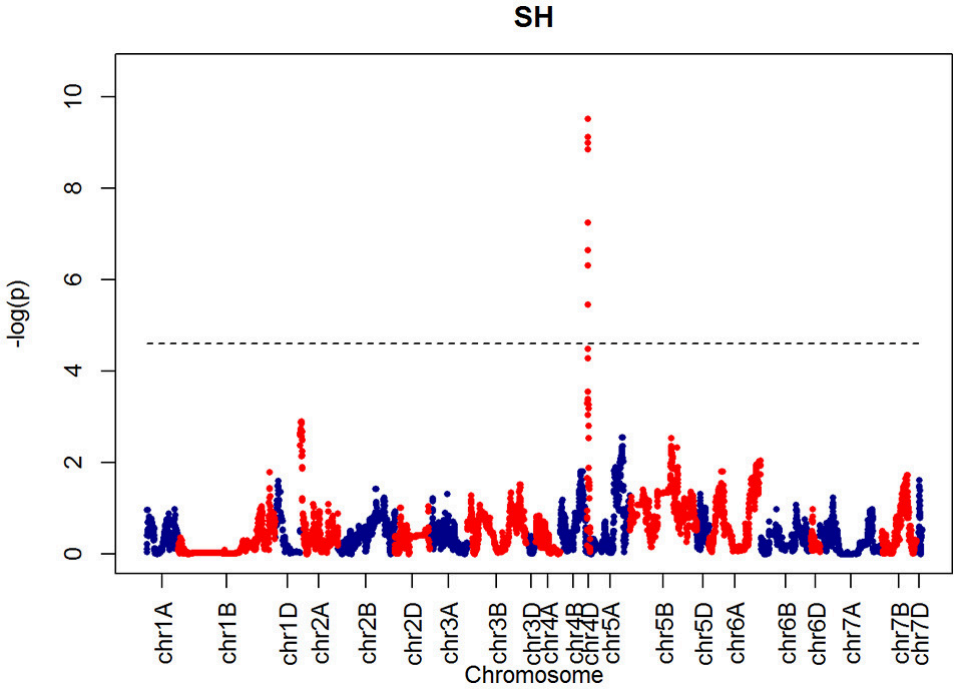
**QTLs markers for the trait SH**. Dashed lines indicate threshold (FDR < 0.05, logP4).

**Figure 11:**
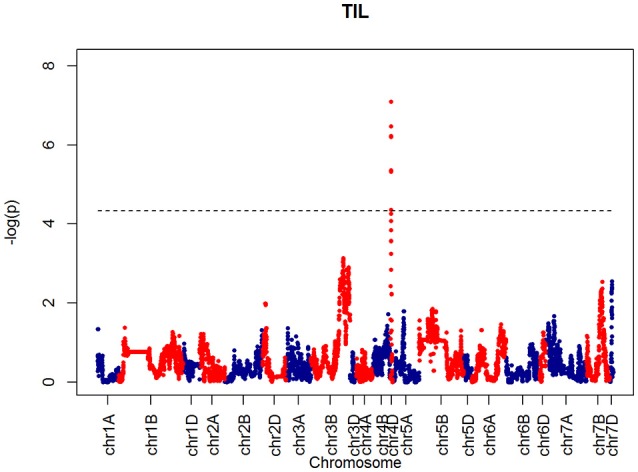
**QTLs markers for the trait TIL**. Dashed lines indicate threshold (FDR < 0.05, logP4).

For SH, one QTL was identified on chromosome 4D at 32.24 cM which corresponded to the marker RAC875_c1673_193 (Figure 10). A QTL for TIL was also identified in the same location (Figure 11). QTLs for OEW and PW were also identified on 4D at 26.97 cM corresponding to RAC875_c6922_291 (Figure S10A) and 4D at 32.24 cM corresponding to RAC875_c6922_291 (Figure S10B), respectively. We identified one QTL for TEW on chromosome 4D at 26.97 cM, corresponding to RAC875_c6922_291 (Figure S10D). All of these QTLs co-located with the semi-dwarfing gene *Rht-D1* (Rht2) (Ellis et al., 2002). The Rht (Reduced height) genes *Rht-B1* (Rht1) or *Rht-D1* (Rht2) are present in many high-yielding, semi-dwarf varieties, where they offer simple genetic control of high harvest index and resistance to lodging (Flintham et al., 1997).

For HI, 1 weak peak QTL on chromosome 2D at 55.4 cM was identified which corresponded to RAC875_c6922_291 (Figure S10E). Figure S9 shows a contrast between FLS and HI across all the MAGIC and elite lines. Cadenza and Soissons have some of the highest HIs and the shortest time to senesce. Table 2 also shows that Soissons has the highest contribution (0.6) to that particular marker.

## Discussion

This study screened a core set of lines derived from the NIAB wheat MAGIC population under Smarthouse conditions as strategy to understand the physical and genetic relationship between different phenological traits. This strategy resulted in the detection of QTL interactions with novel traits suggesting that the methodology should be taken further in the future.

Pair-wise correlation between all traits identified high positive (≥0.69) correlations between GS39, GS55, GS65 and senescence at the flag leaf (FLS), and between TEW and PW. There was also a negative correlation between FLS and length of the first flag leaf (FFLL), indicating that the shorter the flag leaf the more delayed was the start of senescence. Short flag leaves provide less nutrient assimilation therefore the plant has to compensate by either living longer, or producing a large number of tillers. Consistent with this idea, PC analysis also indicated a correlation between these traits. Suggestions that delayed leaf senescence leads to increased yield have been thrown into doubt (Borrill et al., 2015) but it may contribute under certain conditions. It will be interesting to see whether the correlation between leaf length and senescence is maintained under other environmental conditions. Genetically unstructured populations such as the MAGIC collection will be ideal to test whether experimental manipulation of the size of the sink (grain mass) can further modulate flag leaf senescence.

In order to see if there was any clustering of individuals according to phenotype, a projection plot of the MAGIC lines onto the first two PCs was generated (Figure S6). The map shows lines grouped around 3 clusters of traits where Group 1 contained Cadenza, Zircon, Xi-19, Claire, Alchemy, Soissons, and Robigus; Group 2 contained Brompton and Hereward and Group 3 contained Avalon, showed similar trait profiles. Group 1 was early to senescence and group 2 later. Group 3 contained smaller plants as indicated by their opposite location to the FEL, SEL, and TEL vectors.

FLS was also negatively correlated with disease resistance (SM), indicating that highly susceptible plants were more likely to trigger senescence early. Support for this perspective may come from the observation that Xi-19 had the earliest FLS of all the parents and controls (Figure S9) and the joint highest disease score. In the field, Xi-19 flowers considerably later than the earliest flowering parent, Soissons, which carries the *Ppd-D1a* allele for early flowering (Scarth et al., 1985).

After confirming absence of population structure with PCA of the kinship matrix, QTL mapping identified a very strong marker (−logP > 8.00) on chromosome 5A associated with GS39 and GS55, which we believe is likely to correspond to the vernalization gene *VRN-A1*. This gene plays an important role in the vernalization process in diploid (*Triticum monococcum*) and polyploid wheat (*Triticum aestivum*) (Loukoianov et al., 2005; Kiss et al., 2014). However, using HAPPY, no significant QTL was found on chromosome 2D, the location of the *Ppd-D1* locus, for GS39 or GS55. In the field, the presence of the *Ppd-D1* allele in Soissons results in this line flowering 7–14 days earlier than the other MAGIC founder lines. These contrasting results between field and CER for *Ppd-D1* and *Vrn-A1* associated QTL suggest that the plants in this experiment might have experienced reduced vernalization as a result of a lack of cold treatment. However, this idea was discarded because inspection of CER records did not show any temperature discrepancies during vernalization. Another possibility is that plants displayed disease-like symptoms at an early stage. We also noticed that Cadenza, one of the 4 elites and a genotype that does not need vernalization, was one of the first to senesce, had the shortest duration between GS39 and GS55 and was more disease susceptible than the similarly early-flowering Soissons (Figures 4, 5). The fact that Cadenza has no vernalization requirement, might suggest that indeed plants we not fully vernalized or encountered a de-vernalizing effect. Whatever the cause, the results of the experiment appear to have been strongly affected by a vernalization issue. This may explain some of the “anomalous” behavior of Cadenza, Xi-19 and Zircon, all of which do not require vernalization.

Further insight is provided by QTL validation analyses using mpMap. With no covariates included in the QTL model, the mpMap interval mapping approach produces very similar results to HAPPY. However, many more QTLs (−logP > 10) are detected using a model with 10 covariates in mpMap, as can be seen in Figure S11. For GS39 and GS55, it can be seen that although the 5A QTL is still the highest peak, the *Ppd-D1* marker is significant and detected as the third (GS39) or 2nd (GS55) highest QTL. For FLS, 5A is also the most prominent QTL, but there is no evidence for a QTL around the *Ppd-D1* locus. This supports the observations from the trait analysis that progression through different developmental processes e.g., flowering vs. senescence, is controlled independently. Furthermore, QTL detected using mpMap for the length of the interval (d3) from flowering to senescence (Figure S11C) show a distinct pattern from both GS55 and FLS, although some loci are in common (e.g., 4D). As well as phenological traits, markers associated to morphological traits were also identified. For example, a strong marker on chromosome 4D was associated with shoot height (SH) and shoot number (TIL), as well as ear weight (OEW) and above ground biomass (PW). In all these cases, the QTL interval includes the *Rht-D1* (*Rht2*) locus. The *Rht-D1b* allele at this locus causes a semi-dwarfing phenotype in wheat, is strongly correlated with a reduction in height and several other morphological traits and is segregating in the MAGIC population (dwarfing alleles are present in all parent lines except Robigus and Soissons). Interestingly, this locus also shows up in the highest QTL interval for the d3 developmental interval in the mpMap covariate analysis (Figure S11D), suggesting that there may be independent effect on timing of progression through development. The *Rht-D1b* allele has a premature stop codon resulting in reduced sensitivity to gibberellic acid, which has been associated with reduced plant height and earlier heading date (Wilhelm et al., 2013). Our analysis indicates there are differential effects on the duration of other developmental processes not directly related to height or flowering *per se*.

In addition to FLS, GS55, and GS39, the multiple covariate analysis also identified a strong (−logP > 18) peak on chromosome 7B (Figure S11E). Chromosome 7 has been previously associated to Septoria leaf blotch in an analysis of wheat-barley disomic addition lines. The highest level of resistance to infection by *S. tritici* was found in the *H. vulgare* chromosome addition line 7 followed by 4 and 6 (Rubiales et al., 2001).

Another interesting result is related to HI, for which a weak peak QTL was identified on the 2D chromosome, the location of *Ppd-D1*. In the field, the presence of the *Ppd-D1* allele in Soissons results in flowering 7–14 days earlier than the other MAGIC founder lines. In our analysis, Soissons have some of the highest HIs and the shortest time to senesce but it also has the highest contribution (0.6) to that particular maker.

This study provides the first systematic phenological characterization of a wheat MAGIC population under controlled environment conditions. With careful developmental staging and end of life measurements of the MAGIC core set we were able to identify previously detected QTL loci on chromosomes 5A and 4D associated with the onset of senescence at the flag leaf. This powerful multi-founder population captures much of the genetic variation present in elite cultivars and a more detailed knowledge of fine-scale developmental and physiological patterns can be exploited for fine-tuning wheat's response to the environment. We have shown that the combination of MAGIC + Smarthouse can help extend the current understanding of developmental plasticity in elite wheat varieties with potential application for responding to the adaptation challenges facing agriculture in a changing climate.

## Author contributions

Conceived and designed the study: AB and AC; analyzed the data: AC; assisted with QTL analysis: RM, KG, and IM; provided genetic data: KG and IM; provided scoring data: FC; wrote the paper: AC; provided comments and corrected the manuscript: All authors.

### Conflict of interest statement

The authors declare that the research was conducted in the absence of any commercial or financial relationships that could be construed as a potential conflict of interest. The reviewer FM and handling Editor declared their shared affiliation, and the handling Editor states that the process nevertheless met the standards of a fair and objective review.
