# Supplementary material for: Determining Phenological Patterns Associated with the Onset of Senescence in a Wheat MAGIC Mapping Population
Source: Front Plant Sci. 2016 Oct 24;7:1540. doi: 10.3389/fpls.2016.01540 (PMC5075575; doi:10.3389/fpls.2016.01540)
Supplement: Supplementary file 2 [file DataSheet1.PDF]

Supplementary material

Table S1 Infection type score table

score	description
0	No visible symptoms
1	Small lesion surrounded by necrotic tissue
2	Small lesion surrounded by necrotic tissue
3	Medium sized lesion surrounded only by chlorotic tissue
4	Large lesion surrounded by green tissue

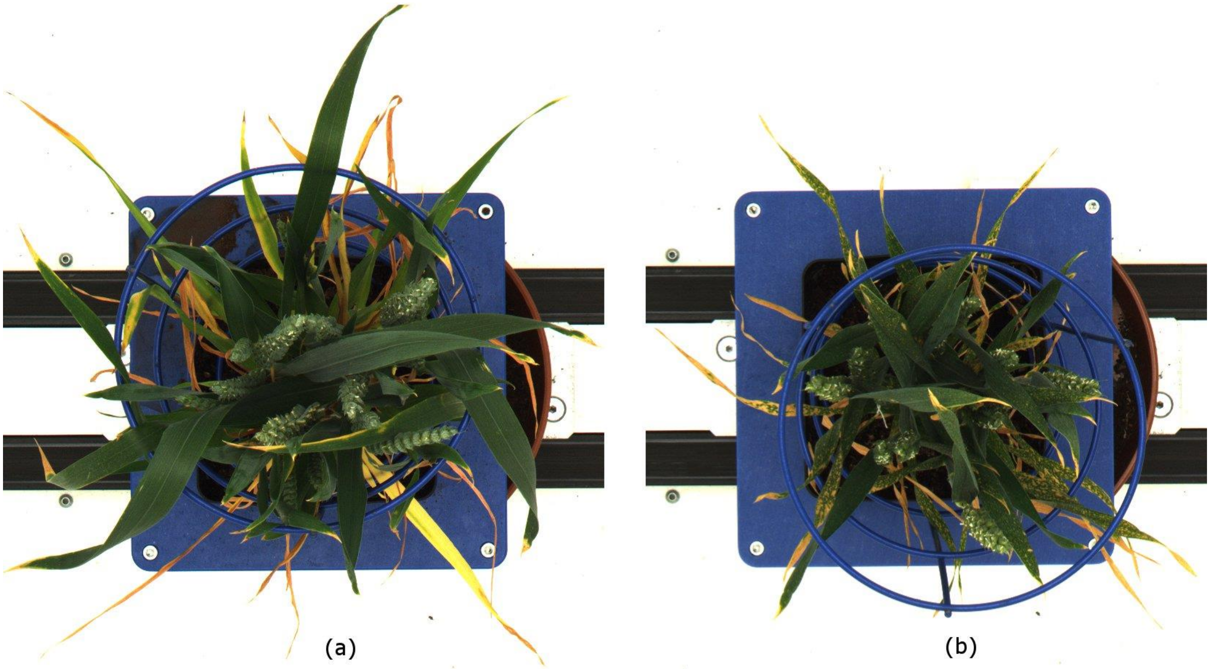

Figure S1 Plant disease symptoms on selected RILs. (a) Score 0. No chlorotic or necrotic lesions. (b) Score 4. Clear signs of lesions on most leaves. Images were taken at 165 DAS.

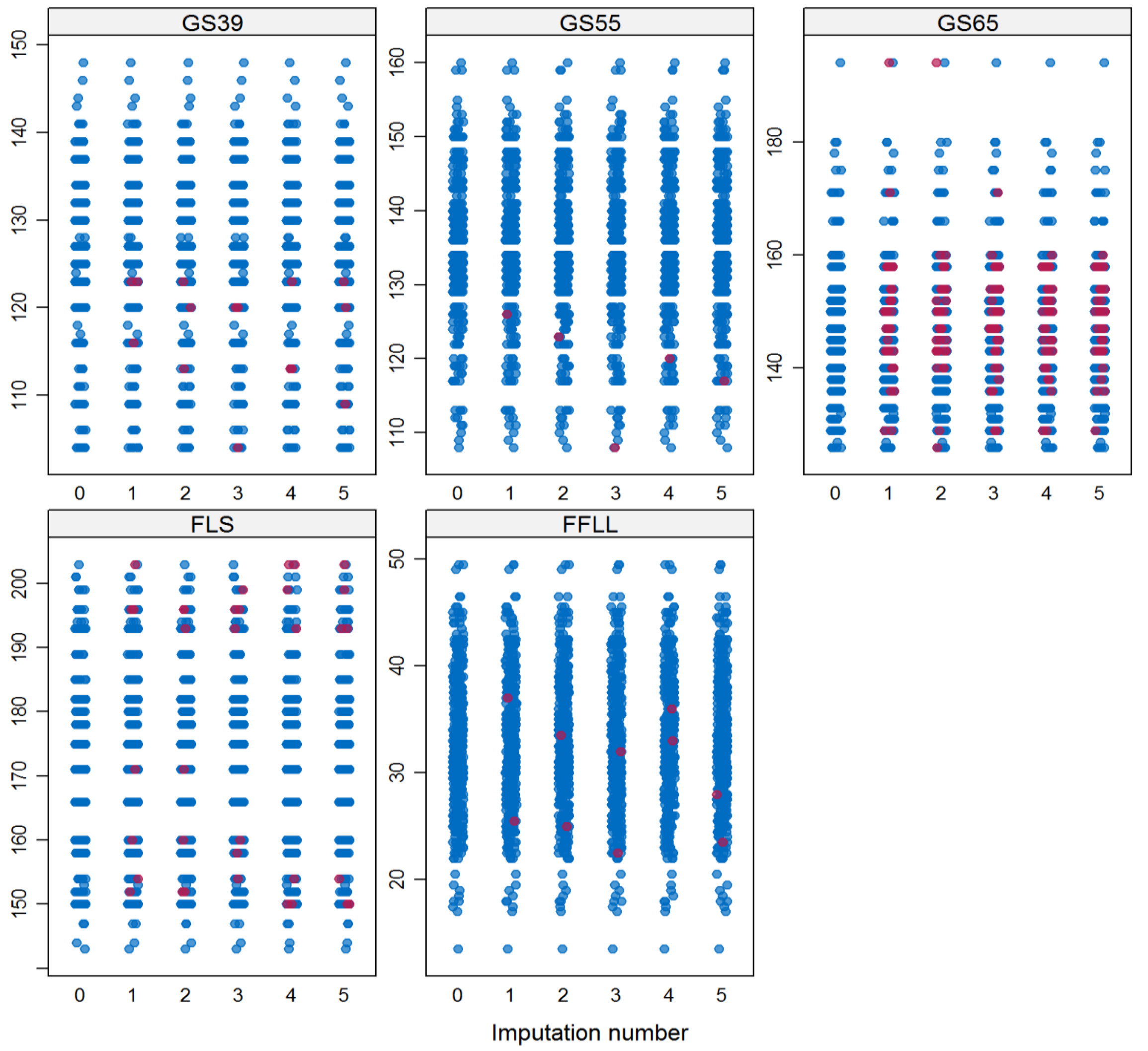

Figure S2 Comparing the distributions of original and imputed data for those traits with missing data. Original data are shown in red, imputed data in blue. The output shows the imputed data for each observation (first column left) within each imputed dataset (remaining five columns).

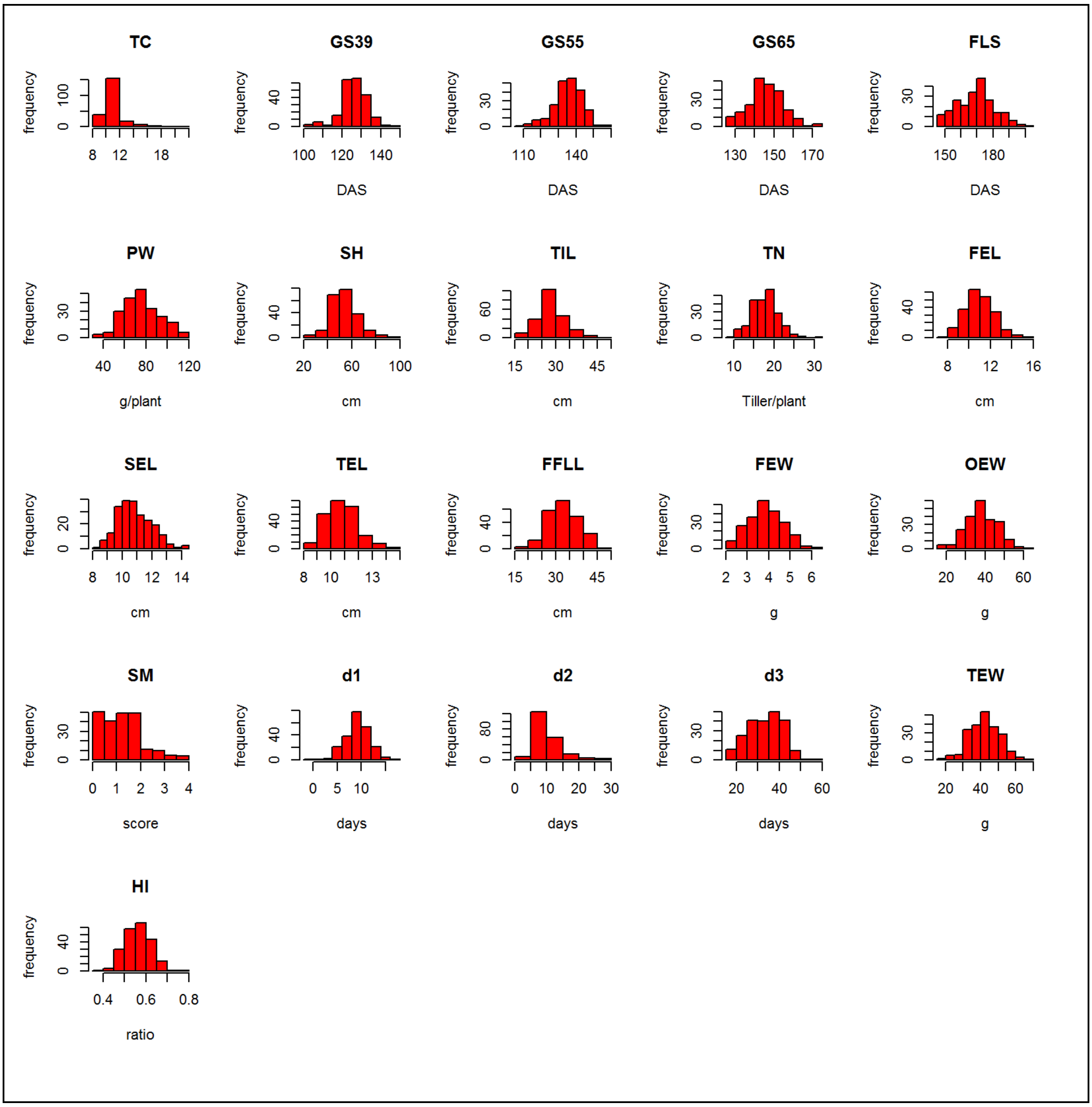

Figure S3 Frequency distribution of all traits for all RILs

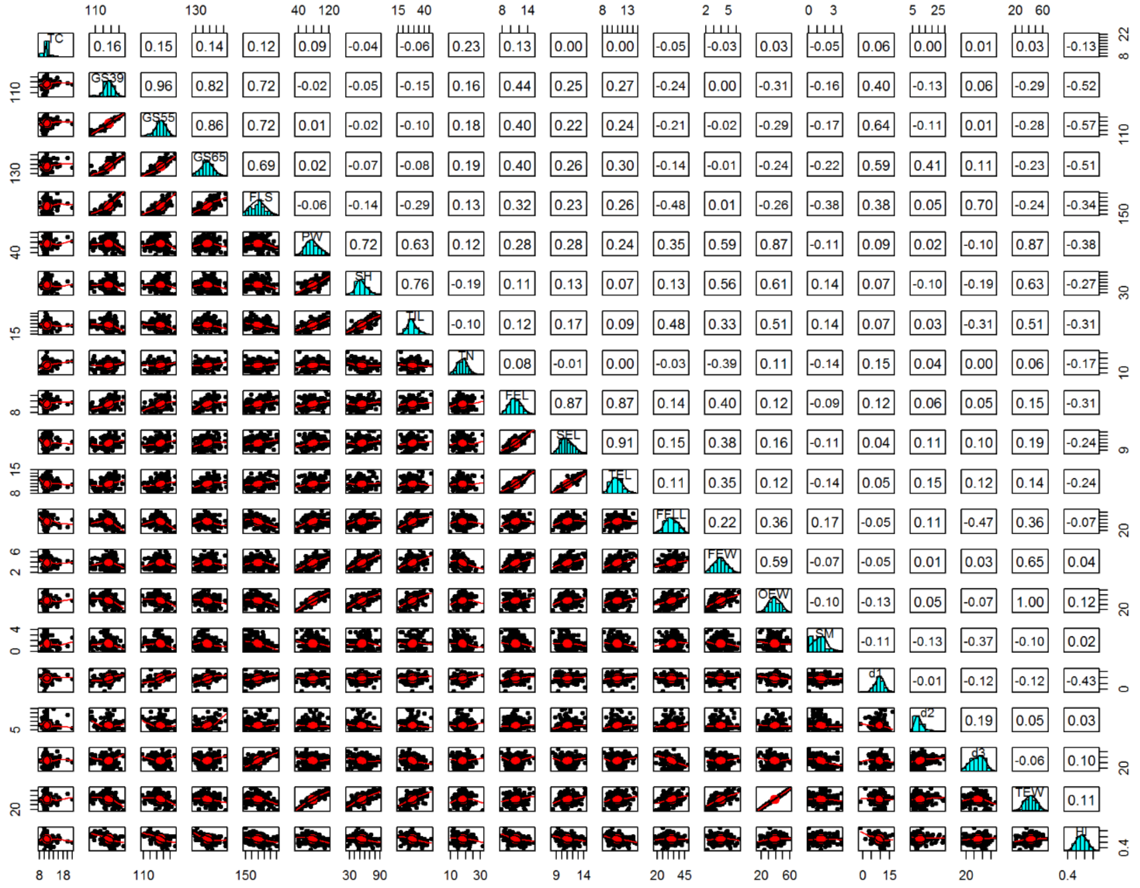

Figure S4 Pair-wise correlation analysis of all traits analysed in this study. Lower panels (below the diagonal) provide a pairwise scatter plot of the traits (provided on the diagonal). For example, first scatter plot corresponds to TC on the vertical axis and to GS39 on the vertical axis. The upper panels indicate the correlation coefficients. Diagonal panel also provides the frequency distribution for each trait value over the population.

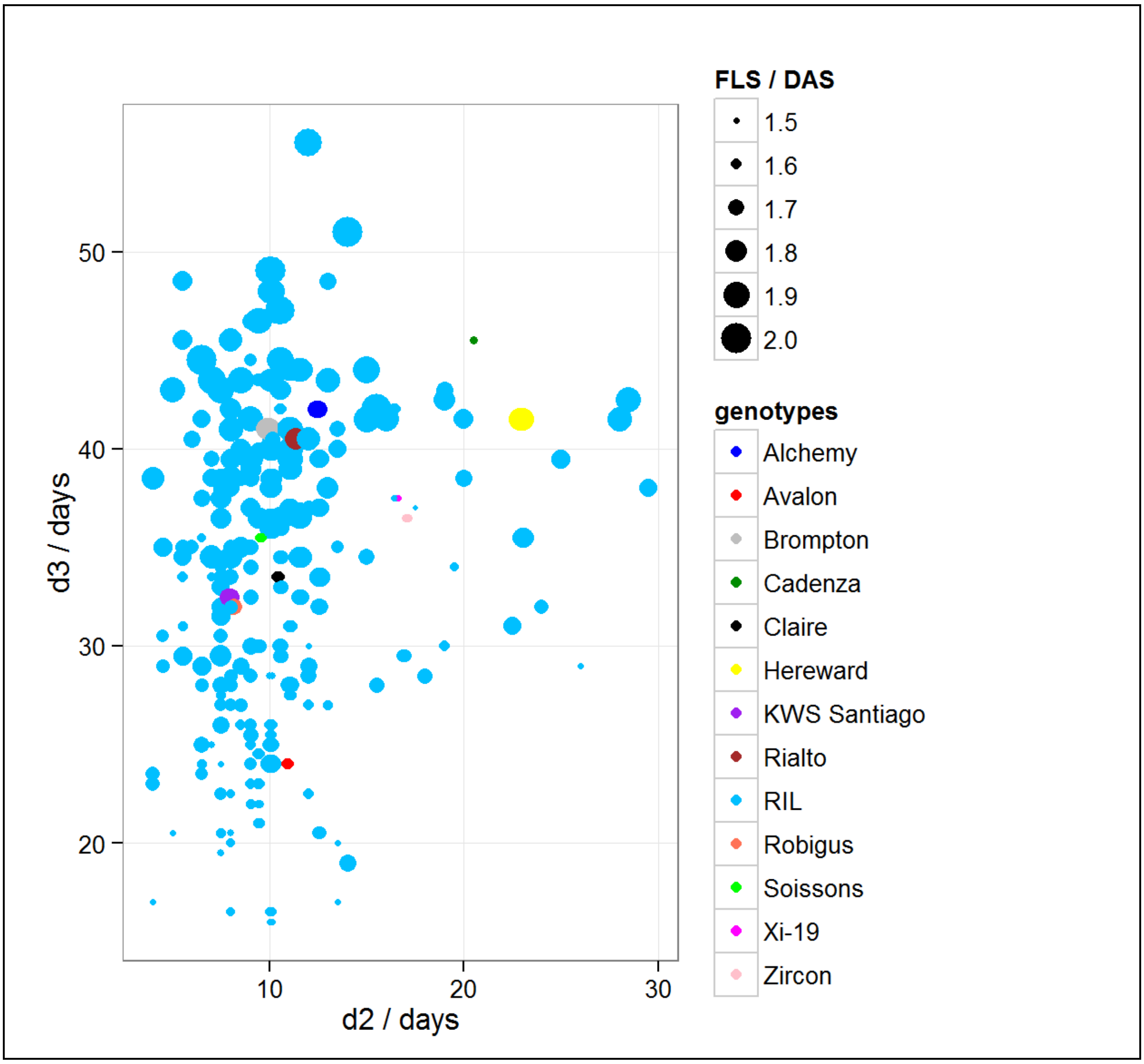

Figure S5. Variation between d2 and d3. Dot size represents number of days to FLS/100 (divided by 100 to be able to show the dots in the plot). Blue dots correspond to RILs. Colour dots correspond to MAGIC parents.

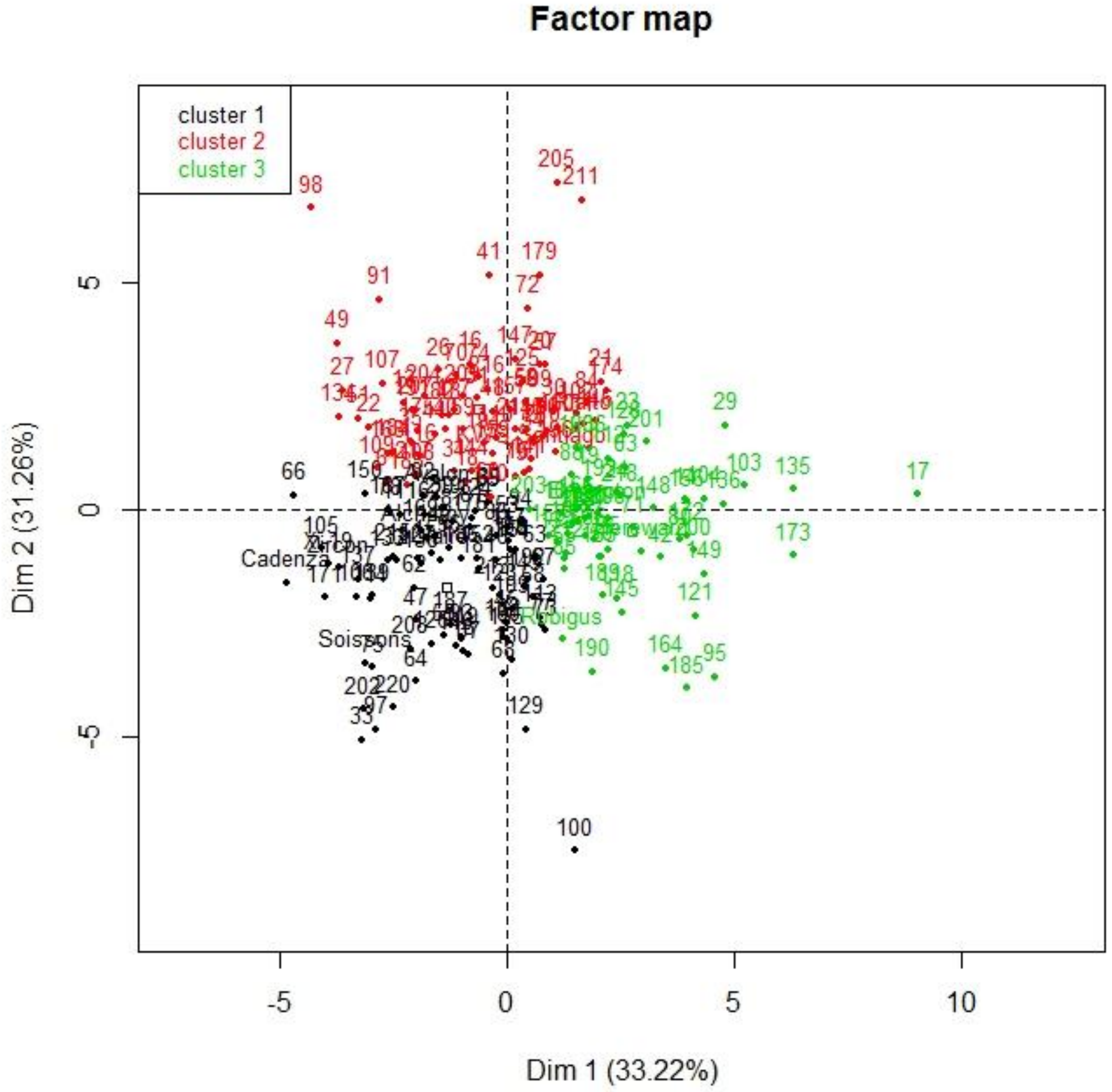

Figure S6. Hierarchical Clustering on Principal Components. Biplot shows a projection plot of the magic lines onto the first two PCs to see if there is any clustering of individuals's phenotypes. MAGIC parents and elite lines are labelled only to facilitate interpretation.

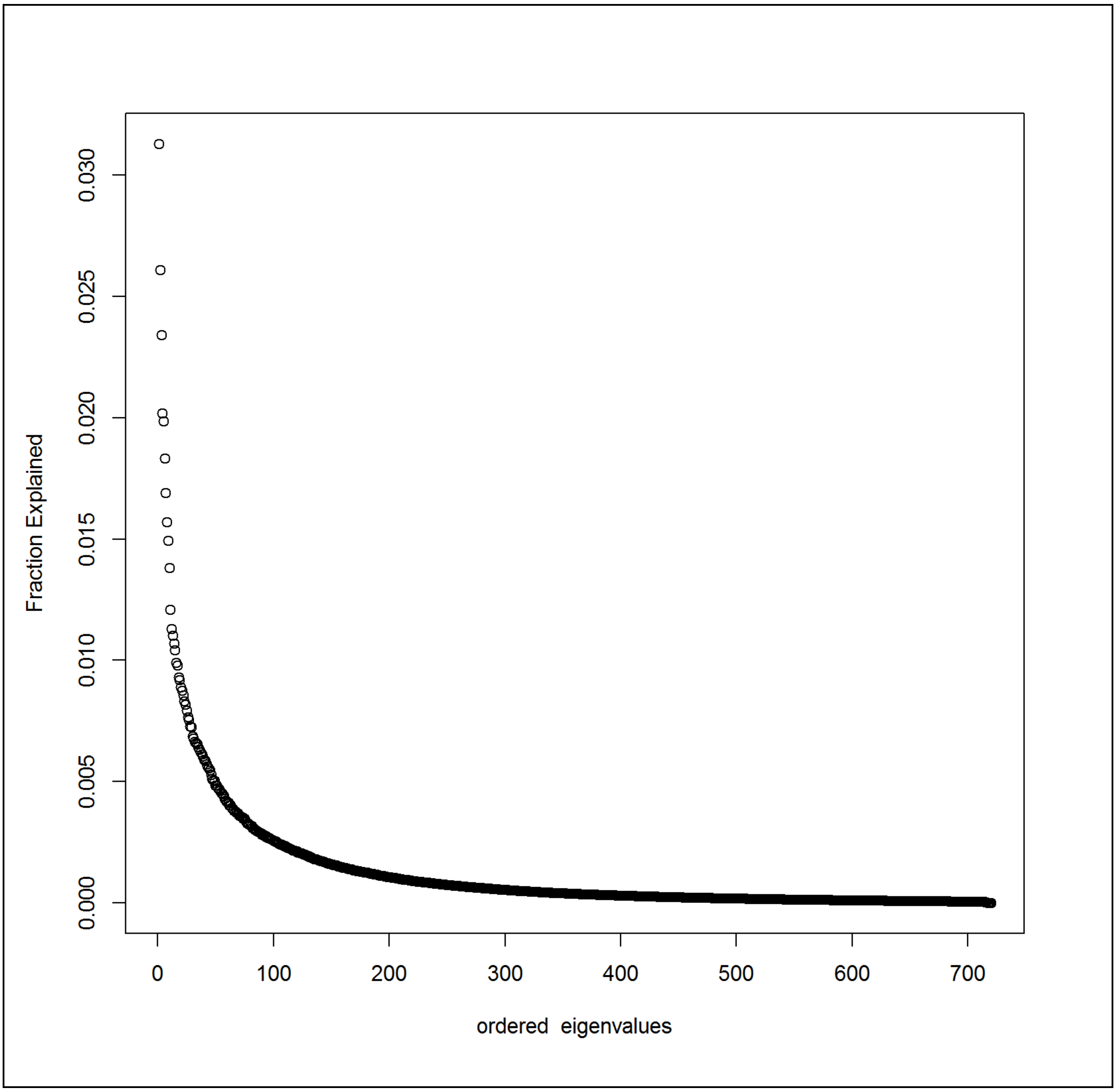

Figure S7. Biplot of a PCA analysis by eigenvalue decomposition of the marker based relationship matrix (A). First PC accounts for less than 4% of the total spectrum..

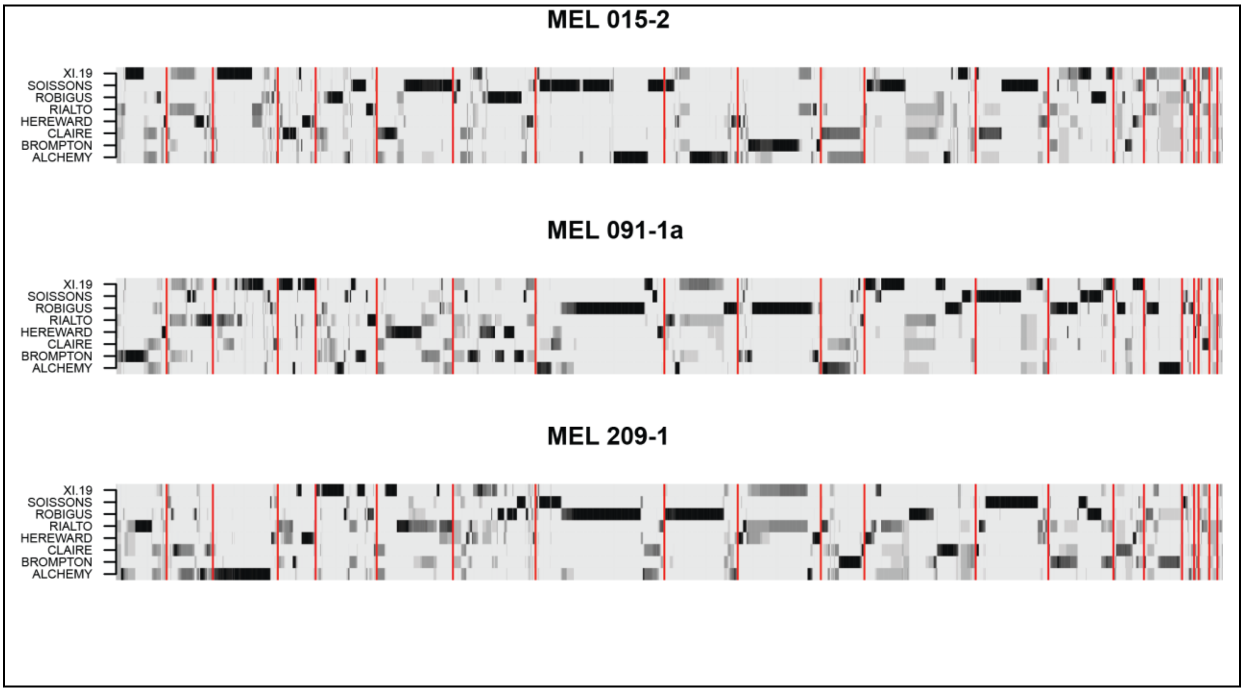

Figure S8. Genomics mosaics of four RILs: MEL 15-2, MEL 091-1a and MEL 209-1. Vertical axis represents the MAGIC parents and horizontal represent chromosomes.

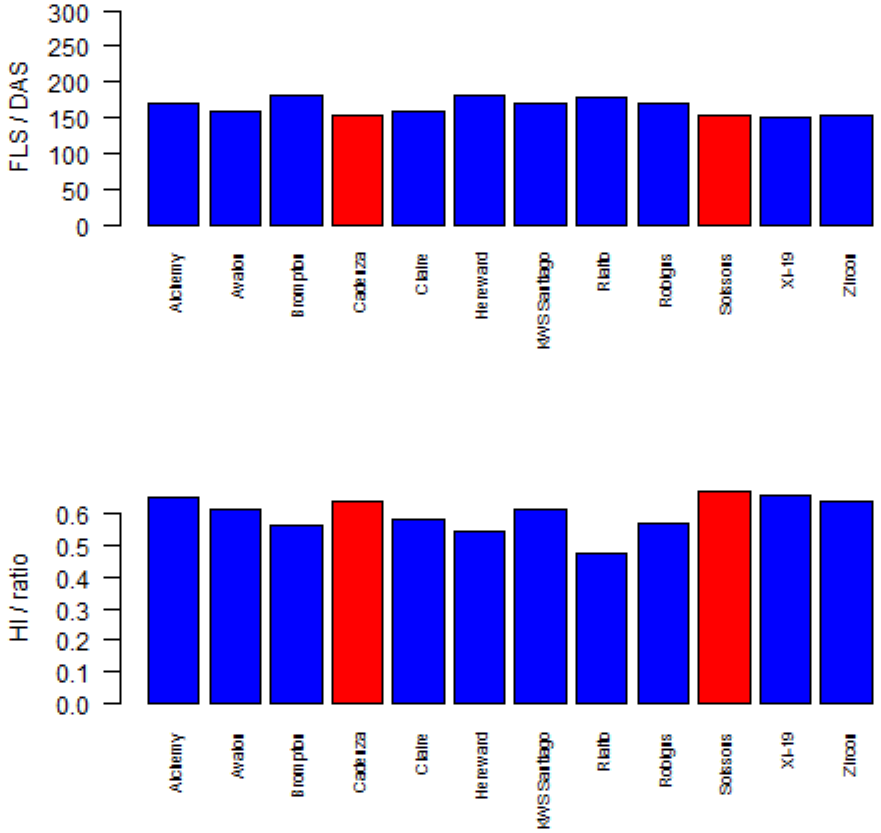

Figure S9. Barplot of FLS and HI across all MAGIC parents. Red bar indicates Cadenza and Soissons. DAS (Days after sowing)

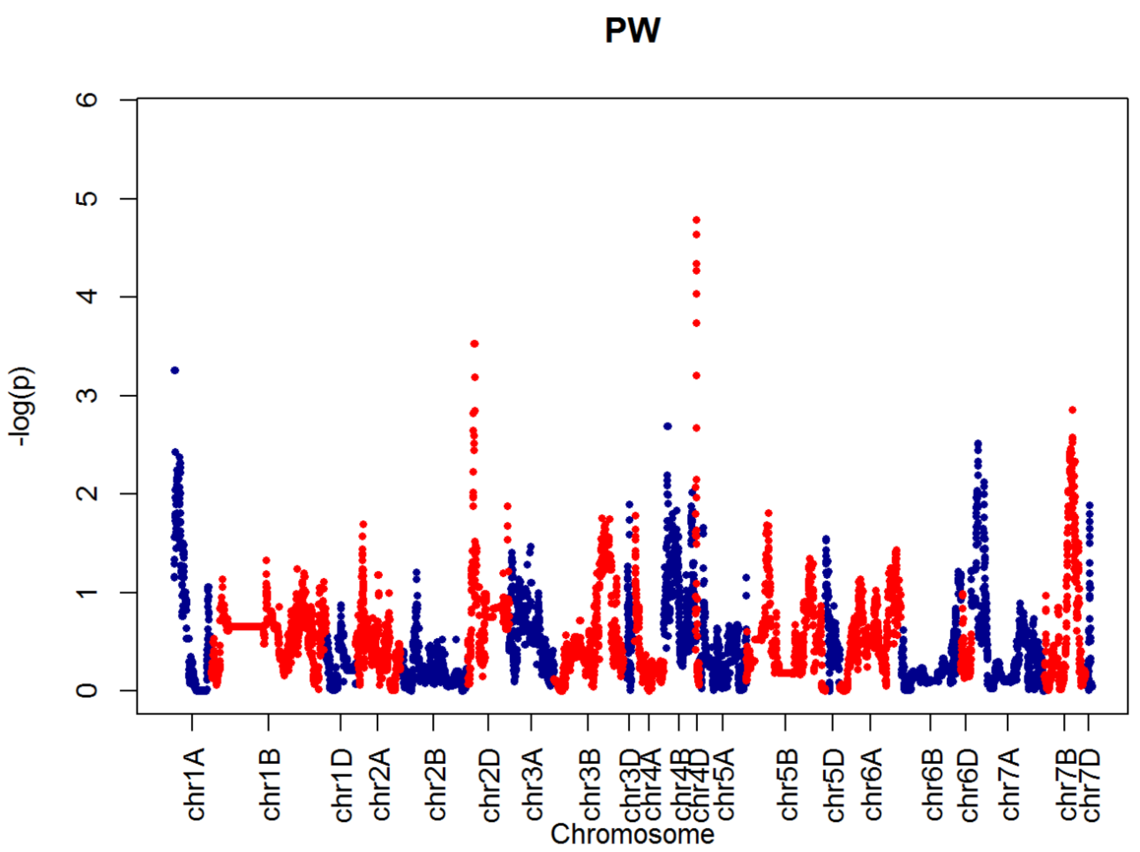
PW
-log(p)
0
1
2
3
4
5
6
chr1A
chr1B
chr1D
chr2A
chr2B
chr2D
chr3A
chr3B
chr3D
chr4A
chr4B
chr4D
chr5A
chr5B
chr5D
chr6A
chr6B
chr6D
chr7A
chr7B
chr7D
Chromosome

(a)

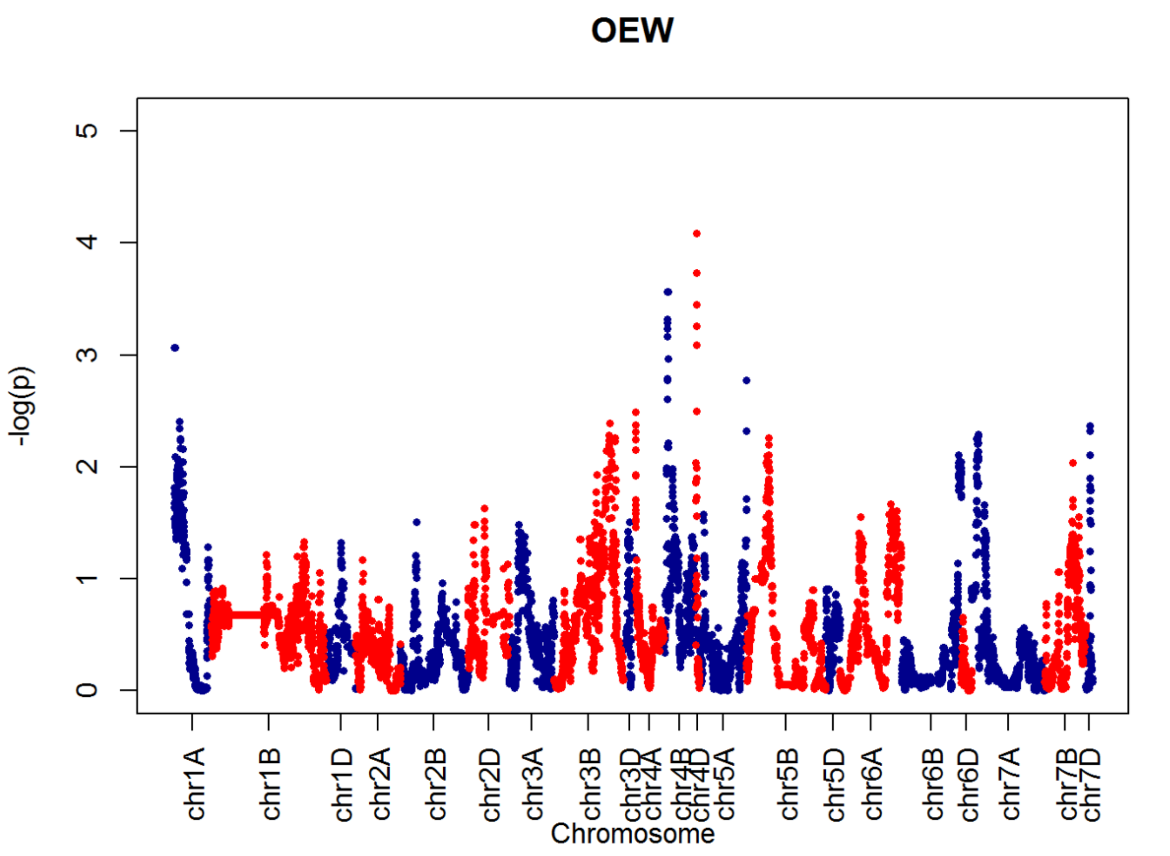
OEW
-log(p)
0
1
2
3
4
5
chr1A
chr1B
chr1D
chr2A
chr2B
chr2D
chr3A
chr3B
chr3D
chr4A
chr4B
chr4D
chr5A
chr5B
chr5D
chr6A
chr6B
chr6D
chr7A
chr7B
chr7D
Chromosome

(b)

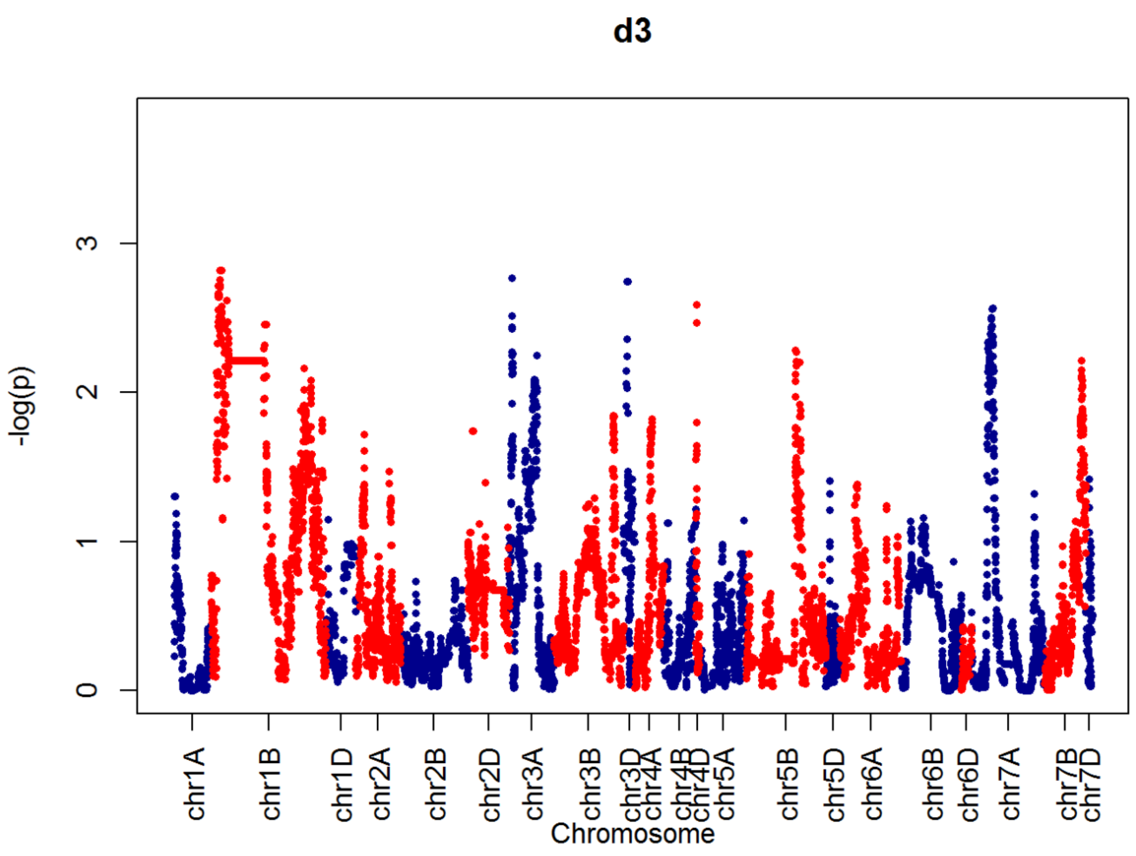
d3
-log(p)
0
1
2
3
chr1A
chr1B
chr1D
chr2A
chr2B
chr2D
chr3A
chr3B
chr3D
chr4A
chr4B
chr4D
chr5A
chr5B
chr5D
chr6A
chr6B
chr6D
chr7A
chr7B
chr7D
Chromosome

(c)

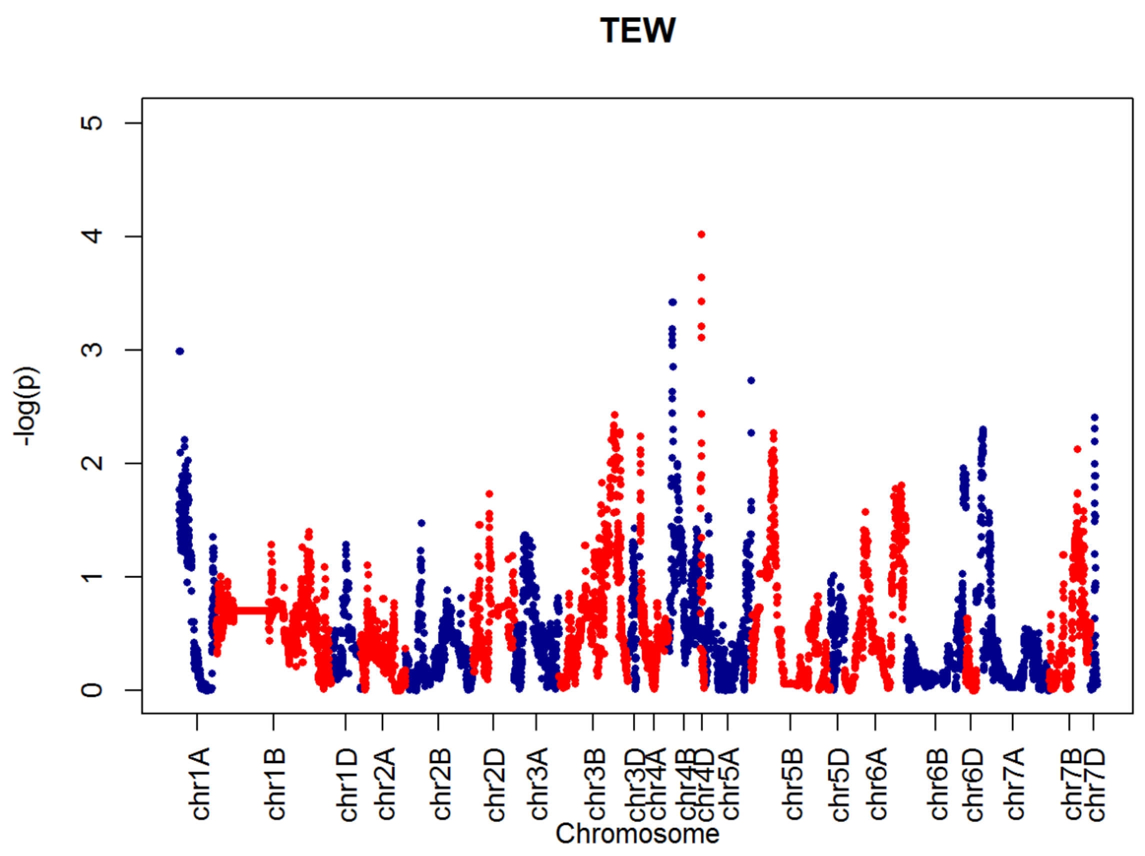
TEW
-log(p)
0
1
2
3
4
5
chr1A
chr1B
chr1D
chr2A
chr2B
chr2D
chr3A
chr3B
chr3D
chr4A
chr4B
chr4D
chr5A
chr5B
chr5D
chr6A
chr6B
chr6D
chr7A
chr7B
chr7D
Chromosome

(d)

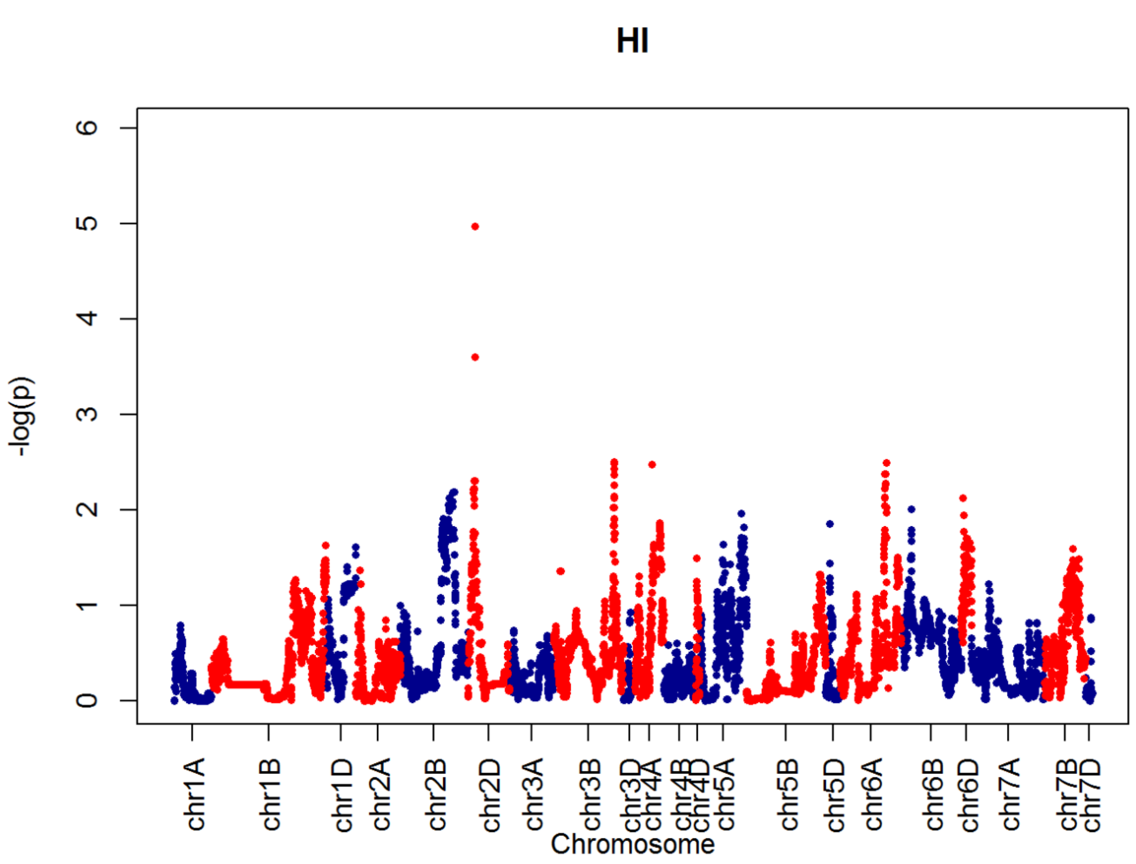

(e)

Figure S10 Manhattan plots for the traits, logP = 4, (a) PW and (b) OEW, (c) d3, (d) TEW and (e) HI.

46

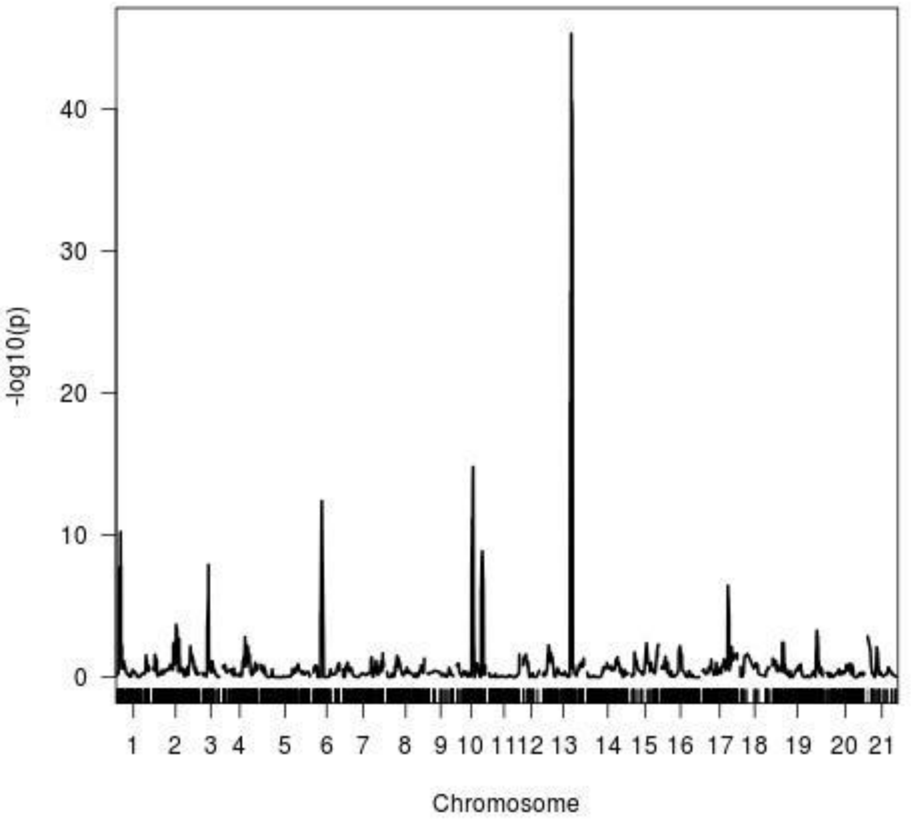
40
30
20
10
0
-log10(p)
1 2 3 4 5 6 7 8 9 10 11 12 13 14 15 16 17 18 19 20 21
Chromosome

(a)

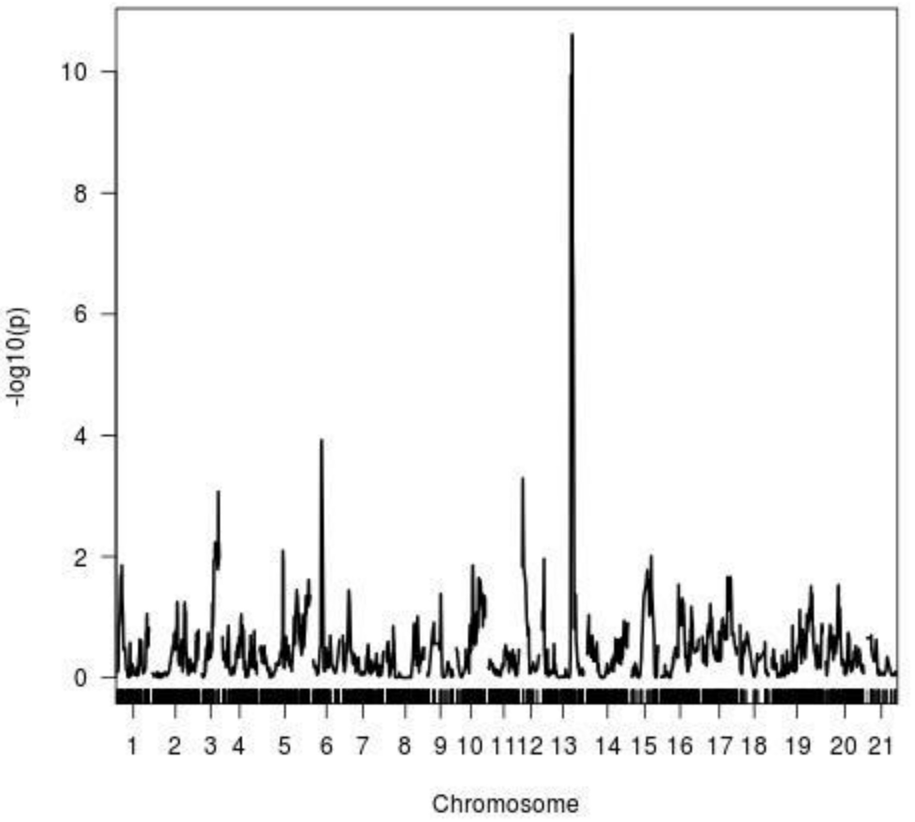

-log10(p)
0
2
4
6
8
10
1 2 3 4 5 6 7 8 9 10 11 12 13 14 15 16 17 18 19 20 21
Chromosome

(b)

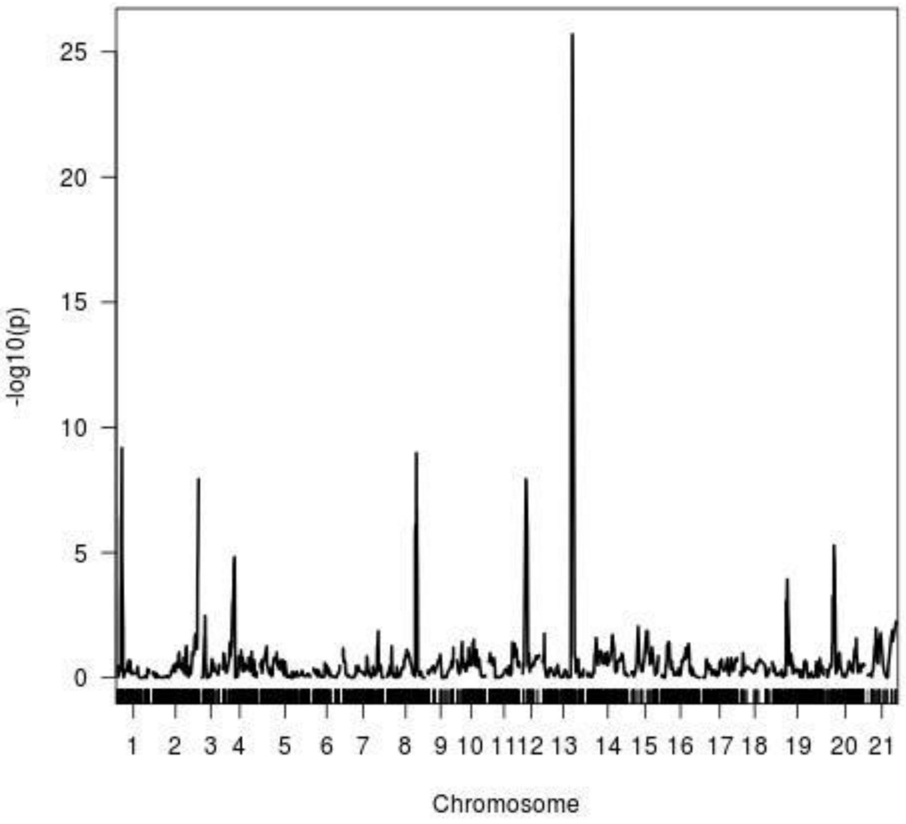
25
20
15
10
5
0
-log10(p)
1 2 3 4 5 6 7 8 9 10 11 12 13 14 15 16 17 18 19 20 21
Chromosome

(c)

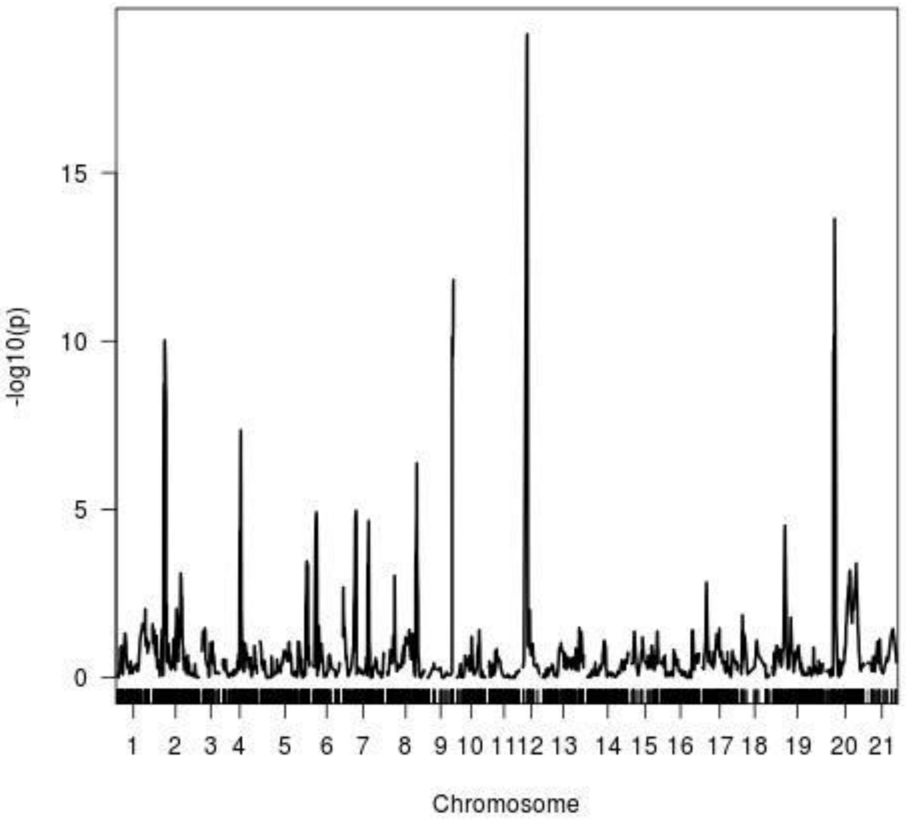

15
10
5
0
-log10(p)
1 2 3 4 5 6 7 8 9 10 11 12 13 14 15 16 17 18 19 20 21
Chromosome

(d)

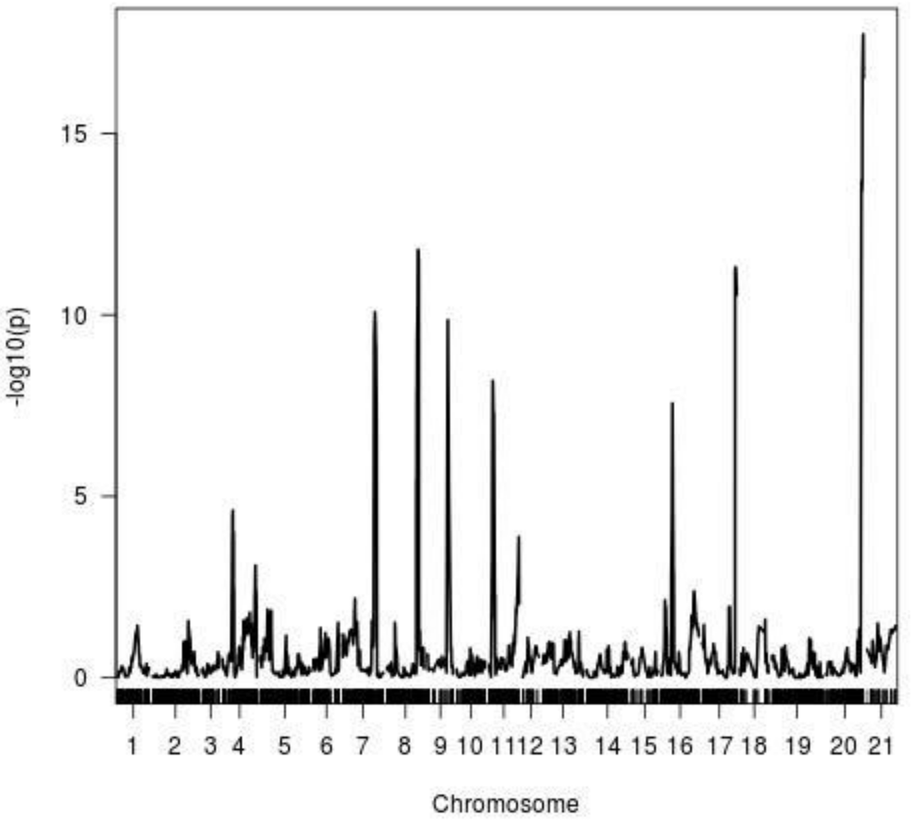

(e)

Figure S11. Scan plot from QTL analysis of (a) GS39, (b) GS55, (c) FLS (d) d3 and (e) SM using 10 covariates, where 1-21 = 1A,1B, 1D, 2A, 2B, 2D, 3A, 3B, 3D ….7D. Plots are produced by the R package mpMap.
